# Associations between work stressors, measured by the demand-control-support and effort-reward-imbalance models, and diurnal cortisol concentrations in saliva

**DOI:** 10.1007/s00420-026-02209-3

**Published:** 2026-09-09

**Authors:** Sigurd Mikkelsen, Julie Lyng Forman, Johan Hviid Andersen, Esben Meulengracht Flachs, Matias Brødsgaard Grynderup, Åse Marie Hansen, Linda Kaerlev, Henrik Albert Kolstad, Reiner Rugulies, Stinna Skaaby, Jane Frølund Thomsen, Jens Peter Bonde

**Affiliations:** 1 Department of Occupational and Environmental Medicine, Bispebjerg and Frederiksberg University Hospital, Copenhagen, Denmark; 2 Section of Biostatistics, Department of Public Health, University of Copenhagen, Copenhagen, Denmark; 3 Department of Occupational and Environmental Medicine, Danish Ramazzini Centre, Goedstrup Hospital, Herning, Denmark; 4 Department of Occupational Medicine, Danish Ramazzini Centre, Aarhus University Hospital, Århus, Denmark; 5 Department of Public Health, University of Copenhagen, Copenhagen, Denmark; 6 National Research Centre for the Working Environment, Copenhagen, Denmark; 7 Psychiatric Center North Zeeland, Copenhagen University Hospital, Capital Region of Denmark Mental Health Services, Hillerød, Denmark; 8 Research Unit of Clinical Epidemiology, Institute of Clinical Research, University of Southern Denmark, Odense, Denmark

**Keywords:** Job-strain, Demand-control-support, Effort-reward-imbalance, Diurnal cortisol pattern, Allostatic load

## Abstract

**Purpose:**

Chronic exposure to work-related mental stressors has been linked to higher risk of disease, possibly mediated by effects on the normal diurnal cortisol concentration pattern due to allostatic overload. The purpose of this study was to examine if the diurnal cortisol pattern changed with exposure to work-related stressors according to the demand-control-support (DCS) and the effort-reward-imbalance (ERI) stress models.

**Methods:**

In 2007, 4467 Danish public service employees participated in a study on job stress and mental health, and 3217 were reexamined in 2009, using identical methods. DCS and ERI-exposures were assessed from self-administered questionnaires. Cortisol concentrations were assessed from saliva samples, one in the morning and one in the evening. Saliva sampling time distributions enabled us to estimate exposure effects on cortisol concentration patterns for the first two hours after awakening and for nine hours in the evening, and on the peak-to-nadir slope. Associations between the exposures and the diurnal cortisol pattern were examined by linear mixed model analyses, separately for DCS- and ERI-exposures, with mutual adjustment for main and composite variables of the stress model and a large set of potential confounders.

**Results:**

DCS- and ERI-exposures were not associated with changes in the diurnal cortisol pattern. In contrast, many associations between potential confounders and cortisol were statistically significant, including the diurnal slope pattern assessed from saliva sampling times.

**Conclusions:**

We found no support for the hypothesis that DCS- or ERI- exposures are associated with changes of the diurnal pattern of cortisol concentrations in saliva samples.

**Supplementary Information:**

The online version contains supplementary material available at https://doi.org/10.1007/s00420-026-02209-3.

## Introduction

Chronic or frequently recurrent exposure to psychological stressors at work has been linked to a range of adverse health outcomes (Karasek and Theorell 1990; Niedhammer et al. 2021; Siegrist and Wahrendorf 2016). A potential mechanism of these relations is stressor-related allostatic overload of the hypothalamic-pituitary-adrenal axis (HPA-axis) and an abnormal pattern of diurnal cortisol concentrations.

The normal diurnal pattern of cortisol concentrations is characterized by a steep increase for 30–45 minutes after awakening to reach its diurnal peak (the cortisol awakening response (CAR)). It then declines exponentially until reaching its nadir approximately 15 hours after waking, followed by an exponential increase until waking up next morning (Adam and Kumari 2009; Karlamangla et al. 2013) .

The allostatic overload theory posits that normal adaptive physiological reactions to stressors may become dysfunctional and cause disease if stressor exposure of a certain magnitude become chronic or frequently repeated over a longer period of time (Chrousos 2009; Juster et al. 2010; McEwen 1998, 2007). The hypothalamic-pituitary-adrenal axis (HPA-axis) normally reacts to acute stressor exposure by a steep increase of cortisol secretion followed by a gradual return to normal after stressor termination. Most healthy people with high reactivity to acute stress habituate to repeated exposure with a reduced or no acute cortisol response (Allen et al. 2014; Foley and Kirschbaum 2010; Manigault et al. 2020). Allostatic overload and dysregulation of the HPA-axis may lead to both hyper-cortisolism and hypo-cortisolism (McEwen 2007; Miller et al. 2007). Furthermore, it is characterized by a change in the normal diurnal cortisol pattern with a flatter morning-to-evening slope and a higher evening concentration (Adam et al. 2017; Johar et al. 2014; Karlamangla et al. 2022; McEwen 2007).

This study examines how diurnal saliva cortisol concentrations vary with exposure to work-related psychological stressors according to the demand-control-support (DCS) model (Johnson and Hall 1988; Karasek and Theorell 1990) and the effort-reward-imbalance (ERI) model (Siegrist 2016). Both models have been associated with increased risk of incident cardiovascular and mental disorders with allostatic overload of the HPA-axis as a proposed mechanism (Siegrist and Wege 2020; Steptoe and Kivimaki 2013).

The DCS model posits that high psychological demands is a stressor if job control is low. This combination is referred to as job-strain. The model predicts that adverse health effects result from the interaction of job demands and job control (Karasek 1979; Karasek et al. 1988). The model further posits that low support at work increases adverse effects of job-strain. The combination of high job-strain and low support is referred to as iso-strain (Johnson and Hall 1988; Karasek and Theorell 1990).

The ERI model posits that high efforts and low rewards are independent stressors, and that the ratio between increasing effort and decreasing reward (ER-ratio), has stronger adverse health effects than the separate effects of efforts and rewards. The model further posits that high levels of a personal coping trait, overcommitment, has a separate adverse effect on health and increases adverse effects of the ER-ratio. The term ‘imbalance’ refers to the concept of ‘failed reciprocity’ where ‘cost’ (effort) exceeds ‘gains’ (rewards), reflected in an ER-ratio > 1 (Siegrist 2016).

Thus, both stress models consider effects of three main exposures, one composite variable combining two main exposures and one composite variable combining all main exposures, and posit specific effect interactions between main exposures.

Findings in previous studies on associations between work-related stressors, including DCS- and ERI-exposures, and measures of the diurnal cortisol pattern (levels and slopes) have been inconsistent with relatively few significant findings in opposing directions (Boggero et al. 2017; Eddy et al. 2023; Karlson et al. 2012; Siegrist 2016). Previous studies have generally been limited by small sample sizes and little exposure contrast (Boggero et al. 2017; Eddy et al. 2023; Karlson et al. 2012; Siegrist 2016). Most studies only examined effects of job-strain or the ER-ratio with no adjustment for confounding effects of main exposures or of the three-way composite exposures (Alves et al. 2013; Lang et al. 2022).

The aim of the present study was to examine if DCS- and ERI-stressors would change the diurnal cortisol pattern according to the allostatic overload hypothesis. We hypothesized that there would be no such changes.

This study examines the mutually adjusted effects of all components of each of the two stress models on the diurnal pattern of cortisol concentrations in saliva samples in a large cohort of Danish municipality workers.

## Methods

### Study population

In January 2007, we invited 10,036 public sector employees in Aarhus, Denmark to participate in a study on stress and mental health, the PRISME-study. The study population worked in almost 400 different work units and consisted of hospital staff (mainly nurses, nursing assistants and medical doctors), social work and counseling professionals and teachers. About 5% were blue collar workers (Kolstad et al. 2011). The baseline and follow-up distribution by age, sex, education, income and other descriptive factors are presented in Results “Potential confounders”section.

Participants were asked to fill in a questionnaire on working conditions, health, and other personal factors, to collect saliva in two sampling tubes, one in the morning and one in the evening, and to record sampling circumstances in a saliva sampling log. In January 2009, respondents from 2007 were asked to participate in a repetition of the baseline study. A total of 4486 participants returned saliva sample material (saliva tubes and sampling logs), 4.467 at baseline, 3.217 at follow-up, and 3.198 on both occasions. Time differences between examination round responses were on average 24 months (SD 0.7), range 20–27 months.

### Cortisol and saliva sampling times

Due to funding shortage, we could only afford two cortisol samples per participant and examination round. We asked participants to collect one saliva sample 30 minutes after awakening and one at approximately 20:00 the same evening, preferably on a workday, and to record the sampling time on the sample label. We did not ask for behavioral pre-sampling restrictions (e.g., tooth brushing, smoking, eating etc.) as such requirements could reduce participation. The instruction emphasized that swabs should be kept in the mouth until thoroughly saturated and then in a refrigerator until they were returned by mail. Dates of sample receipt for analyses were recorded. Samples were stored at – 20 °C and analyzed within 10 months. Cortisol concentrations in saliva were analyzed with the Spectria Cortisol Coated Tube RIA (Orion Diagnostica, Finland) by the same laboratory at both examination rounds. Further methodological details have been published previously (Garde et al. 2003; Vammen et al. 2014).

Participants were asked to fill a saliva sampling log on waking time on the saliva sampling day, bedtime the night before, morning and evening sampling times, and weather samples were taken on a workday or a day off. All times were recorded by date, hours and minutes.

**Fig. 1:**
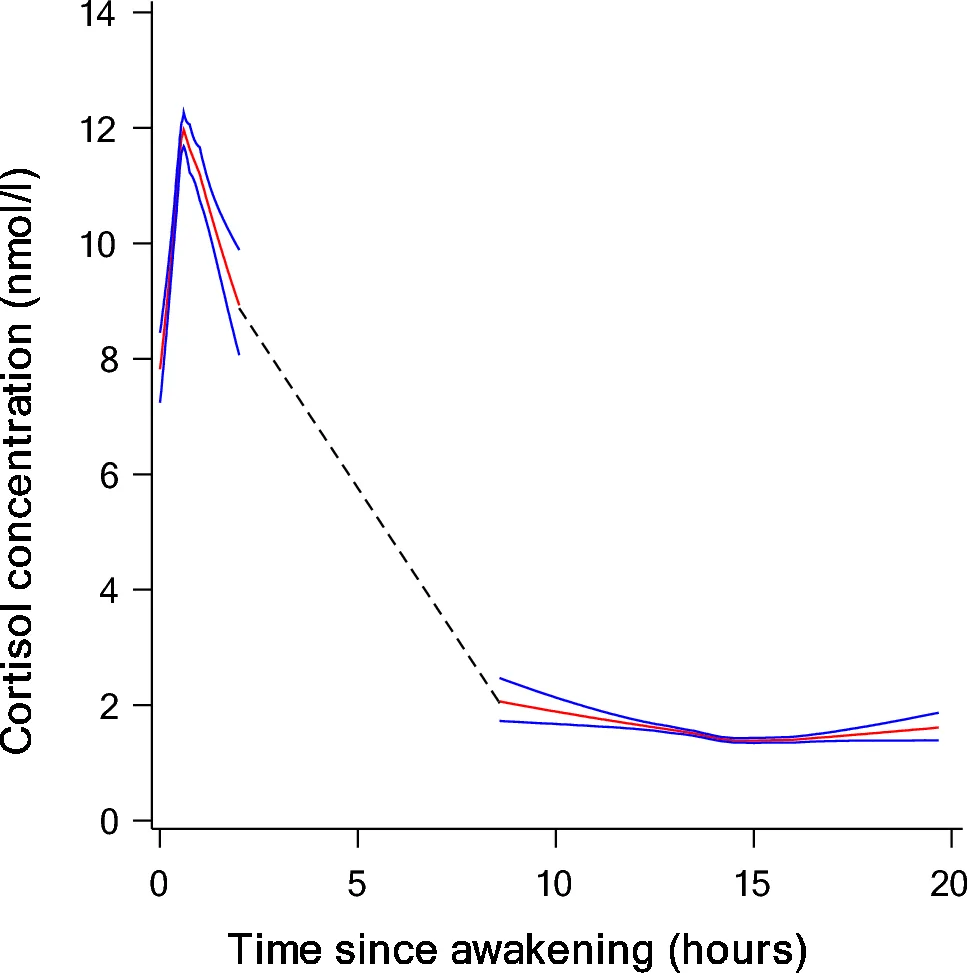
**Cortisol concentrations by time since awakening.** Estimated geometric means (red lines) and their 95% confidence limits (blue lines) The straight dashed black line is an interpolation line connecting the estimated geometric means of morning and evening cortisol. Loess analyses (smooth option=0.7). Saliva sampling times since awakening were 0 to 2 hours in the morning (n=5978) and 8.6 to 21.5 hours in the evening (n=6672). No adjustment for potential confounders. Evening saliva sampling times were transformed to time since awakening by subtracting awakening time from the sampling time.

Consistency of waking times and saliva sampling dates and times was reviewed across saliva sampling tube labels, sample logs, and laboratory receipt dates. We excluded samples with inconsistent or missing data, sampling intervals exceeding 24 hours, and sample transit times (days from saliva sampling to laboratory receipt) exceeding eight days. Morning samples were considered valid if taken between 03:00 and 12:00 and within 2 hours from awakening. Evening samples were considered valid if taken between 17:00 and 02:30. We chose wide time frames to increase the precision of estimates of diurnal cortisol slopes and levels and to accommodate irregular work schedules. Cortisol concentrations above 100 nmol/l were excluded. There was a considerable variation in saliva sampling times around the times recommended in the instruction letter. Owing to the large sample size, most 5–10-minute intervals of morning samples and 15–30-minute intervals of evening samples were represented by several hundred samples (Table 1). This variation allowed assessment of mean cortisol variation with time from waking until two hours later, from 17:00 until 02:30, and population peak-to-nadir slopes. When evening sampling times were specified relative to waking time, the raw diurnal cortisol pattern (Fig.1) was in good accordance with findings in other studies, e.g., Karlamangla et al. (2013).

**Table 1:** Number of valid cortisol saliva samples (n) by saliva sampling times with associated geometric means and 95% confidence intervals (CI) of cortisol concentrations (nmol/l)^a^

Morning				Evening			
Saliva sampling times (minutes from waking)	n	Cortisol (nmol/l)		Saliva sampling times (clock time hh:mm)	n	Cortisol (nmol/l)	
		Geometric mean	95% CI			Geometric mean	95% CI
0– < 5	181	8.2	7.4–9.3	17:00–19:29	219	2.1	1.9–2.3
5– < 15	142	8.6	7.6–9.8	19:30–19:44	238	1.8	1.7–2.0
15– < 20	294	9.7	8.9–10.5	19:45–19:59	403	1.4	1.3–1.5
20– < 25	121	11.2	9.9–12.5	20:00–20:14	2546	1.5	1.5–1.5
25– < 30	301	10.9	10.1–11.8	20:15–20:29	549	1.5	1.4–1.6
30– < 35	2595	11.8	11.5–12.1	20:30–20:44	489	1.5	1.4–1.6
35– < 40	311	12.3	11.4–13.3	20:45–20:59	205	1.3	1.2–1.5
40– < 45	466	11.9	11.2–12.6	21:00–21:29	548	1.3	1.2–1.4
45– < 50	615	11.5	10.9–12.1	21:30–21:59	287	1.3	1.1–1.4
50– < 60	152	11.7	10.6–12.9	22:00–22:14	329	1.3	1.2–1.5
60– < 65	309	11.2	10.5–12.0	22:15–22:44	287	1.4	1.3–1.6
65– < 80	183	10.8	9.7–12.0	22:45–23:14	264	1.5	1.3–1.6
80– < 95	168	10.2	9.2–11.3	23:15–23:59	293	1.6	1.4–1.8
95–120	140	9.3	8.3–10.4	24:00–02:30	15	1.7	1.1–2.6
Total	5978			Total	6672		

^a^The distribution by examination round is shown in Supplementary material S4

The morning cortisol pattern may be described by the morning cortisol peak and the pre- and post-peak slopes, and the evening pattern by the evening nadir and the pre- and post-nadir slopes. The pre-peak slope is a measure of the CAR. The identity between the two measures is a property of linear growth models: the mean of the individual slopes is the same as the population mean slope. The post-peak slope represents the first steeper part of the post-peak cortisol decline. The pre-nadir slope represents the late flatter part of this decline, and the post-nadir slope represents the first flatter part of cortisol increase from nadir to waking.

Slopes were estimated as cortisol change per time unit of two-piece linear splines of morning and evening sampling times with peak- and nadir-times as inflexion points. Peak and nadir times were predefined based on non-linear regression analyses from a a previous study of a comparable dataset (Mikkelsen et al. 2017). Peak-time was 34.2 minutes (0.57 hours) after waking at both examination rounds, and nadir time was 20:46 at baseline and 21:14 at follow-up.

### DCS- and ERI-exposures

DCS- and ERI-exposures were measured by self-administered questionnaires.

*Demands, control and support* were measured with items from the Copenhagen Psychosocial Questionnaire (Kristensen et al. 2005). Demands, decision authority and skill discretion were each assessed by four items, and support by two items. Each item had five verbally anchored response categories ranging from ‘always’ to ‘never/almost never’ or ‘seldom/never’, scored 1–5. Scale scores were calculated as the mean of item scores.

*Effort, reward and overcommitment* were measured with 16 items from the short form ERI questionnaire (Siegrist et al. 2009) in a standardized Danish translation (Eller et al. 2012; Weyers et al. 2006). Effort was assessed by three items, reward by seven items and overcommitment by six items. Each item consisted of a statement on a work-related situation that might be stressful. Effort and reward items were scored by the original two-step response option format (Siegrist et al. 2009).

The first response option was to agree or disagree with a statement indicating work stress (scored 1 if disagreed with); if agreed with, the respondent was asked to rate the associated level of distress (‘does not bother me’, ‘it bothers me a little’, ‘it bothers me somewhat’ and ‘it bothers me a lot’, scored 2–5). Overcommitment items were rated on a four-point Likert scale from ‘strongly disagree’ to ‘strongly agree’, scored 1 to 4. Scale scores were calculated as the means of item scores.

If necessary, item and scale scores were reversed to express increasing stress at work (high demands, effort and overcommitment; low control, support and reward). Scale scores was set to missing if half or more scale items were unanswered. Item wording and scoring details are provided in Supplementary material S1.

#### Continuous composite exposures

Job-strain was defined as the product of demands and control, and iso-strain as the product of job-strain and support. The ER-ratio was defined as the ratio between effort and increasing reward, and the interaction of the ER-ratio with overcommitment (O-ER-ratio) was defined as the product of the ER-ratio and overcommitment. Product terms were divided by the maximum score of the last variable to get the number of scale units approximately comparable to the main exposures. Thus, continuous job-strain and ER-ratio vary from 0.20 to 5.0, iso-strain from 0.04 to 5.0, and O-ER-ratio from 0.05 to 5.0. This adjustment was made to facilitate assessment of relative effect sizes of main and interaction terms.

#### Categorical main and composite exposures

Exposures were categorized using questionnaire-based verbal anchors rather than distribution-based cut-points. This approach enhances transparency and interpretability, as verbal categories correspond directly to meaningful response options and facilitate interpretation of exposure-response relationships in the context of allostatic load.

Categories of all main variables were classified as ‘low’, ‘medium’ and ‘high’. Except for support, the ‘medium’ category was defined by scale scores 2.5–<3.5, and ‘low’ and ‘high’ categories by lower and higher scale scores. Categories were ordered to reflect increasing stress (high categories of control, support and reward then reflect low control, support and reward). The medium category for support was defined as scale scores 3.5–4.5 to align verbal anchors to those of demands and control (see Supplementary Material S1).

Categorical two-way composite exposures of demands and control (job-strain) and effort and reward (ER-strain) were categorized by four levels of increasing interaction potential: (1) ‘very low’ (‘low’ category of one exposure and any category of the other), (2) ‘low’ (‘medium’ category of both exposures), (3) ‘medium’ (‘medium’ category of one exposure and ‘high’ category of the other), and (4) ‘high’ (‘high’ category of both exposures). This categorical composite variable was termed ‘ER-strain’ because it was not based on effort-reward ratios.

Categorical iso-strain was defined as job-strain when support was ‘low’ (scale score ≥ 3.5). The categorical three-way composite variable of effort, reward and overcommitment (O-ER-strain) was defined as ER-strain with ‘high’ overcommitment (scale score ≥ 3.5), and else categorized as ‘very low’.

### Potential confounders

Potential confounders were selected from factors associated with diurnal cortisol concentrations or with DCS- or ERI-exposures. Information on date of birth and gender was obtained from administrative registers. Age was calculated as the difference between dates of birth and completing the general questionnaire. From this questionnaire we included information on pregnancy (yes/no), socioeconomic factors, lifestyle, health, sleep quality, and usual work schedule. Socioeconomic factors included vocational education after school (< 3 years, 3–4 years, > 4 years) and personal income (low (< 300,000 DKK), medium (300,000–< 500,000 DKK), high (≥ 500.000 DKK)). Lifestyle included smoking (present smoker (yes/no)), weekly alcohol consumption (> 14 units of ≈ 12.5 g of alcohol (yes/no)), leisure time physical activity (high/low)), and body mass index (BMI = weight (kg)/height (m^2^)). Health aspects included general health (excellent or very good/good/fair or poor), ever diagnosed with a depression or anxiety disorder (yes/no) and ever diagnosed with a cardiovascular disease (yes/no). Sleep quality was measured as ‘disturbed sleep’ during the last 4 weeks (continuous scale, increasing by scores 1-5, Karolinska Sleep Questionnaire (Hansen et al. 2012). Usual work schedule was measured as regular daytime versus other schedules.

From the saliva sampling log*,* we included sampling dates, waking time, sleeping hours (difference between bedtime the evening before and waking time in the morning on the sampling day), and whether samples were taken on a workday or a day off.

Waking time was considered as a continuous variable split into two linear splines with inflexion point at 06:16, identical for both examination rounds, to account for different effects on cortisol of early versus late awakening (Edwards et al. 2001). Age was included as a continuous variable, split into two linear splines at 50 years of age to account for different linear associations in younger and older age groups. Effects of age were assessed per 10 years. BMI was included as a continuous variable with effects assessed per 5 BMI-units. Disturbed sleep and sleeping hours were included as recorded with effects assessed per one scale unit and one sleeping hour, respectively. Sample transit time was considered as a categorical variable (1–4 days versus 5–8 days).

### Statistical analyses

All analyses were made separately for DCS- and ERI-exposures.

The associations of morning and evening cortisol patterns with DSC- and ERI-exposures were assessed by a linear mixed model with the natural logarithm of cortisol concentrations as outcome and with effects of saliva sampling times modeled by two-piece linear splines with pre-defined inflexion times at peak- and nadir-times. Exposure variables, their interactions with saliva sampling time-splines, and potential confounders were included as fixed effects. A random effect of study participant was included to account for repeated measurements from the two examination rounds. Peak-to-nadir cortisol slopes were analyzed using a similar linear mixed model with the differences between the natural logarithms of the morning and evening cortisol concentrations as outcome and exposure variables, pre- and post-peak and pre- and post-nadir time splines, and potential confounders included as fixed effects. Model specifications for the main analyses are shown in Supplementary material S2.

Results are presented as back-transformed estimates with 95% confidence intervals. In analyses of morning and evening cortisol, effect estimates then become the ratio of change in cortisol by a unit change of continuous exposures or of a specified exposure category compared to a reference with low stress exposure. In analyses of peak-to-nadir slopes the exposure effects become the ratio-change in the ratio between morning and evening cortisol concentrations.

The inclusion of interaction terms between exposures and time-splines in analyses of morning and evening cortisol implies that main effects of exposures depend on the reference time point which was set to the peak- and nadir-times, respectively. Main effects of exposures therefore refer to the peak and nadir cortisol concentrations. Exposure effects on morning and evening slopes and the morning-to-evening slope are independent of spline reference times (see Supplementary material S2). Associations were examined in parallel analyses of continuous and categorical exposures.

The null hypothesis of no exposure effects on morning and evening cortisol patterns was assessed by a joint test for any effects on peak or nadir concentrations and associated slopes, considered together. Note that the null hypothesis implies that there is no exposure effect on the area under the curve and no hypo- or hyper-cortisolism associated with the exposure.

Prior to the main analyses, we tested if cross-sectional and longitudinal effects on cortisol outcomes were different for exposures and time-varying confounders, using the method described by (Fitzmaurice et al. 2011). Overall, there were no systematic differences in the cross-sectional and longitudinal effects of exposures or potential confounders (Supplementary material S3). We therefore decided to make no further distinction between cross-sectional and longitudinal effects, which allowed us to assess the effects of exposures with increased statistical power without loss of important information. Since the distinction between cross-sectional and longitudinal effects was not maintained, our results reflect a mixture of their effects.

*Sensitivity analyses* were made to address potential overadjustment by excluding firstly potential confounders which could be antecedent or intermediate factors for the relation between exposures and cortisol (lifestyle, health, sleep factors, and time of awakening), and secondly all potential confounders except examination round (Supplementary material S7). We further examined if collinearity between exposure variables had attenuated their effects on the diurnal cortisol pattern (Supplementary material S8). Finally, we examined exposure effects on the waking-to-nadir cortisol slope by setting the reference time for the morning saliva time-splines to waking time (Supplementary material S2).

No adjustment for multiple testing was performed owing to the post hoc exploratory nature of the study. Therefore, results should be interpreted cautiously as spurious findings cannot be ruled out.

All analyses were performed with SAS version 9.4 (SAS Institute Inc, Cary, NC, USA).

## Results

### Population characteristics and variable distributions

#### Cortisol

A total of 5978 (78%) observations had valid morning cortisol data (concentrations, waking, saliva sampling and transit times). The corresponding figure for evening data (concentrations and evening sampling times) was 6672 (87%) observations. The latter figure dropped to 6230 (81%) in regression analyses which also required valid waking and morning saliva sampling times. A total of 5811 (76%) observations had valid information on both morning and evening cortisol data.

Summary statistics for cortisol concentrations by saliva sampling times is shown in Table 1 and Fig. 1 (and by examination rounds in Supplementary material S4) Transit time average was 5 days (SD=2.6 days)

#### DCS- and ERI-exposures

Internal consistency of scale items (Cronbach’s alpha) for demands, skill discretion, decision authority and support were 0.86, 0.74, 0.76 and 0.70, respectively; and for effort, rewards and overcommitment 0.76, 0.76 and 0.81.

Spearman correlations between main exposure scales varied between 0.06 and 0.56 but were much higher between composite variables and their constituent variables, e.g., 0.80 between demands and job-strain and 0.92 between effort and the ER-ratio (Supplementary material S5).

Summary statistics for the continuous-scale and categorized DCS- and ERI-exposures at baseline and follow-up are shown in Table 2. Distribution statistics indicate slightly lower exposure levels at follow-up. These differences may be due to selection, a shift in perceptions of exposures, an improved work environment or regression towards the mean. Approximately 25% reported high levels of demands and effort; less than 10% reported low levels of control, support and rewards; approximately 3% reported high overcommitment, and < 1% reported high levels of job-strain, iso-strain, ER-ratio and O-ER-ratio. The frequency of an ER-ratio > 1 was 27% at baseline and 22% at follow-up, and the corresponding figures for an O-ER-ratio > 1 were 11% and 8% (not shown in the table). Additional analyses showed that intra-individual exposure scale scores were quite stable with few differences of more than one scale score unit between the two examination rounds (demands 7%; control 1%; support 19%; effort 13%; reward 9%; overcommitment 2%).

**Table 2:** Distribution of continuous DCS- and ERI-exposure scores. Means, standard deviations (SDs), score categories, and missing values. By examination round

DCS-Exposures^a^	Examination round	Means and SDs		Score categories^b^						Missing	
				Low (1–< 2.5)		Medium (2.5–< 3.5)		High (3.5–5)			
		Mean	SD	n	%	n	%	n	%	n	%
Demands	baseline	2.86	0.83	1313	29.9	1931	43.9	1151	26.2	72	1.6
	follow-up	2.82	0.83	941	31.8	1248	42.2	766	25.9	262	8.1
Control	baseline	2.53	0.57	1962	44.7	2174	49.5	255	5.8	76	1.7
	follow-up	2.44	0.55	1510	51.1	1325	44.9	119	4.0	263	8.2
Support	baseline	2.29	0.97	3628	82.9	622	14.2	124	2.8	93	2.1
	follow-up	0.63	0.44	2903	99.8	7	0.2	0	0.0	307	9.5

				Low (0–< 2.5)		Medium (2.5–< 3.5)		High (3.5–5)		Missing	
				n	%	n	%	n	%	n	%
Job-strain	baseline	1.45	0.56	4165	94.9	210	4.8	13	0.3	79	1.8
	follow-up	1.38	0.53	2856	96.7	94	3.2	3	0.1	264	8.2
Iso-strain	baseline	0.71	0.49	4322	99.3	28	0.6	2	0.0	115	2.6
	follow-up	0.63	0.44	2903	99.8	7	0.2	0	0.0	307	9.5

ERI-Exposures^a^	Examination round	Means and SDs		Score categories^b^						Missing	
				Low (1–< 2.5)		Medium (2.5–< 3.5)		High (3.5–5)			
		Mean	SD	n	%	n	%	n	%	n	%
Effort	baseline	2.80	1.05	1817	41.8	1356	31.2	1173	27.0	121	2.7
	follow-up	2.58	1.01	1498	51.0	859	29.2	583	19.8	277	8.6
Rewards	baseline	2.27	0.79	2828	64.5	1202	27.4	353	8.1	84	1.9
	follow-up	2.18	0.90	1988	67.4	676	22.9	285	9.7	268	8.3
Overcommitment	baseline	2.30	0.60	2594	59.1	1631	37.1	167	3.8	75	1.7
	follow-up	2.24	0.57	1886	64.0	982	33.3	77	2.6	272	8.5

				Low (0–< 2.5)		Medium (2.5–< 3.5)		High (3.5–5)		Missing	
				n	%	n	%	n	%	n	%
ER-ratio	baseline	0.83	0.49	4283	98.9	41	0.9	6	0.1	137	3.1
	follow-up	0.77	0.52	2880	98.4	35	1.2	12	0.4	291	9.0
O-ER-ratio	baseline	0.52	0.40	4301	99.8	7	0.2	3	0.1	156	3.5
	follow-up	0.47	0.41	2897	99.6	11	0.4	2	0.1	308	9.6

Row totals add up to N = 4467 (100%) for baselined data and to N = 3217 (100%) for follow-up data

^a^Exposure scores:

Demands, control, support, effort and reward: scale scores (1–5), Overcommitment: scale scores (1–4), Job-strain: scale scores 0.20–5, Iso-strain: scale scores 0.04–5, ER-ratio: scale scores 0.20–5, and O-ER-ratio: scale scores 0.05–5. Exposure scores increase with increasing demands, effort, overcommitment, job-strain, iso-strain, ER-ratio, and O-ER-ratio, and with decreasing control, support, and reward. The directions indicate increasing stress according to DCS and ERI models. Verbal score anchors are described in the Methods section and Supplementary material S1

^b^Note that score categories of composite exposures in this table are based on their continuous scores defined by multiplication of their constituent variable scores as described in the previous footnote, – not to be confused with the composite exposure categories in Table 3

The distributions of the four-category composite exposures are shown in Table 3. The joint distribution of demands and control and of effort and reward distributions defining the four-category definitions of composite variables is shown in Supplementary material S6. The frequency of ‘high’ levels of composite strain variables was low (< 5%) but considerably higher than the corresponding continuous multiplicative variables (Table 2). This was also the case for ‘medium’ levels of strain. These differences were expected due to the different definitions.

**Table 3:** Distribution of composite DCS and ERI exposure categories in analyses of categorical effects on cortisol outcomes

DCS categorical composite variables	Category by increasing strain levels^a^								Total
	Very low		Low		Medium		High		
	n	%	n	%	n	%	n	%	n
Job-strain	4633	63.1	1501	20.5	1089	14.8	118	1.6	7341
Iso-strain	6571	90.5	284	3.9	352	4.9	55	0.8	7262

ERI categorical composite variables									
ER-strain	5368	74.0	644	8.9	884	12.2	360	5.0	7256
O-ER-strain	6016	83.3	320	4.4	603	8.4	281	3.9	7220

^a^Job-strain and ER-strain: ‘very low’ (a ‘low’ category of one exposure and any category of the other); ‘low’ (a ‘medium’ category of both exposures); ‘medium’ (a ‘medium’ category of one exposure and a ‘high’ category of the other); and ‘high’ (a ‘high’ category of both exposures)

Iso-strain and O-ER-strain: as for job-strain and ER-strain if support is ‘low’ (scale score ≥ 3.5) or overcommitment is ‘high’ (scale score ≥ 3.5), respectively, and otherwise classified as ‘very low’

#### Potential confounders

Summary statistics for potential confounders are shown in Table 4. Compared to baseline, participants at follow-up reported higher educational level and income, more daytime work, better general health, more leisure time activity and less smoking. Except for personal income, these changes were generally small and may reflect a positive selection related to higher socioeconomic status. A marked increase in personal income may reflect a 9% salary increase from 2007 to 2009 (Statistics_Denmark 2009).

**Table 4:** Distribution of potential confounders at baseline and at follow-up

Potential confounders	Baseline		Follow-up		Missing at baseline		Missing at follow-up	
Continuous variables	Mean	SD	Mean	SD	n	%	n	%
Age	44.5	10.2	47.0	10.0	0^a^		0^a^	
Waking time (hh:mm)	06:23	00:59	06:30	01:02	347	7.8	257	8.0
Morning saliva sample time (min. after waking)	38	19	38	20	380	8.5	280	8.7
Evening saliva sample time (hh:mm, minutes)	20:38	69	20:52	79	108	2.4	94	2.9
Sleep duration (hours, minutes)	7 h 15 min	1 h 0 min	7 h 18 min	1 h 0 min	376	8.4	284	8.8
Disturbed sleep (scale, scores 1–5)	2.35	0.75	2.29	0.73	62	1.4	73	2.3
Body Mass Index (weight(kg)/height(m^2^)	24.2	4.0	24.5	4.1	96	2.1	89	2.8

Categorical variables	n	%	n	%	n	%	n	%
Saliva samples taken on a day off (yes)	505	11.7	548	17.7	166	3.7	120	3.7
Regular day-time work schedule (yes)	2785	64.4	2053	70.8	145	3.2	319	9.9
Gender (female)	3502	78.4	2525	78.5	0^a^		0^a^	
Pregnant (yes)	138	3.1	76	2.4	0^b^		0^b^	
Education after school								
< 3 years	881	20.0	504	16.1	57	1.3	78	2.4
3–4 years	3033	68.8	2193	69.9				
> 4 years	496	11.3	442	14.1				
Personal income								
Low	2379	53.9	1076	36.2	56	1.3	248	7.7
Medium	1790	40.6	1636	55.1				
High	242	5.5	257	8.7				
Psychiatric disorder, ever (yes)	682	15.3	502	15.6	0^c^		0^c^	
Cardiovascular disorder, ever (yes)	639	14.3	532	16.5	0^c^		0^c^	
General health								
Excellent/very good	2254	51.4	1695	54.0	80	1.8	75	2.3
Good	1616	36.8	1163	37.0				
Fair/poor	517	11.8	284	9.0				
Leisure time physical activity (high)	2061	46.8	1564	50.0	66	1.5	91	2.8
Smoking (present smoker)	794	18.1	435	13.9	72	1.6	95	3.0
Weekly alcohol consumption (high)	367	8.4	275	8.8	93	2.1	102	3.2
Saliva sample transit time								
1–4 days	1895	42.4	1392	43.3	230	5.2	162	5.0
5–8 days	2342	52.4	1663	51.7				

Distribution characteristics refer to responders. Missing percentages refer to the total material (N = 4467 at baseline, N = 3217 at follow-up)

Zero missing: ^a^Age and gender: read from personal identification numbers, ^b^Pregnancy: low questionnaire response rate, indicating the participants may have considered the question as too private. We considered that only a small proportion of non-responders were pregnant and therefore coded all non-responders as non-pregnant. ^c^These disorders were queried about in a list of 16 possible disorders ever diagnosed by a doctor, response options yes and no. However, when the ‘yes’ option was used for one or more disorders, the other disorders were often not answered, and in many cases none of the disorders were ticked with ‘yes’ or ‘no’. We considered that a missing value for specific disorders most likely meant ‘no’, and therefore coded missing values as a ‘no’-response

#### Missing values

The frequencies of missing values of exposures and potential confounders are shown in Tables 2 and 4, respectively. Follow-up was attended by 72% of baseline participants. The frequency of missing values for morning cortisol was 14% and 17% at baseline and follow-up, respectively; for evening cortisol the corresponding figures were 6%. The frequency of morning-to-evening sample time differences exceeding 24 hours was 3.7%. For exposure variables the frequency of missing values at follow-up was approximately 8%.

### Effects of DCS- and ERI-exposures on the diurnal cortisol pattern.

**Fig. 2:**
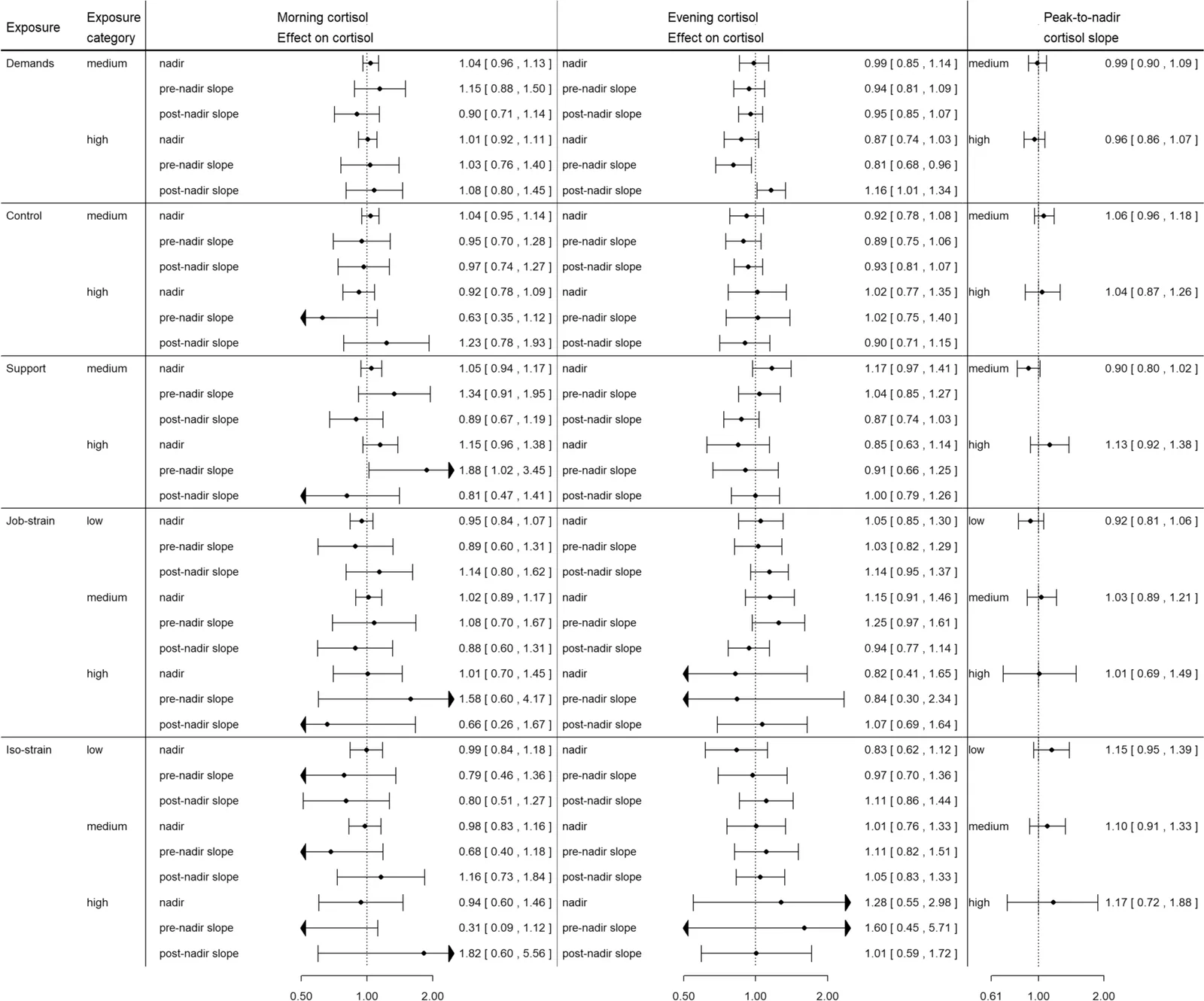
**Forest plots showing estimated effects of DCS categorical exposures on morning and evening cortisol patterns (peak or nadir concentrations with associated slopes) and on the morning-to-evening cortisol slope, mutually adjusted and adjusted for effects of potential confounders.** Effect estimates are the ratios by which the peak or nadir concentrations and associated cortisol slopes change compared to the reference category (‘low’ for main exposures, ‘very low’ for composite exposures). An effect ratio above unity implies that the peak or nadir concentrations increase, that increasing slopes (pre-peak and post-nadir slopes) become steeper, that decreasing slopes (post-peak and pre-nadir slopes) become flatter, and that the morning-to-evening cortisol slope becomes steeper. P-values for the null hypothesis that an exposure has no effect on the morning and evening cortisol pattern (peak or nadir concentrations with associated slopes, considered together) or on the morning-to-evening slope: *Morning cortisol*: demands p=0.80; control p=0.59; support p=0.57; job-strain p=0.88; iso-strain p=0.42. *Evening cortisol*: demands **p=0.02**; control p=0.33; support p=0.06; job-strain p=0.20; iso-strain p=0.77. *Morning-to-evening slope*: demands p=0.77; control p=0.51; support **p=0.04**; job-strain p=0.44; iso-strain p=0.50. Number of observations used in the analyses out of all observations with valid information on cortisol concentrations and saliva sampling times: : morning cortisol n=5105/5978 ; evening cortisol n=5317/6230; morning-to-evening slope n=4969/5811.

**Fig. 3:**
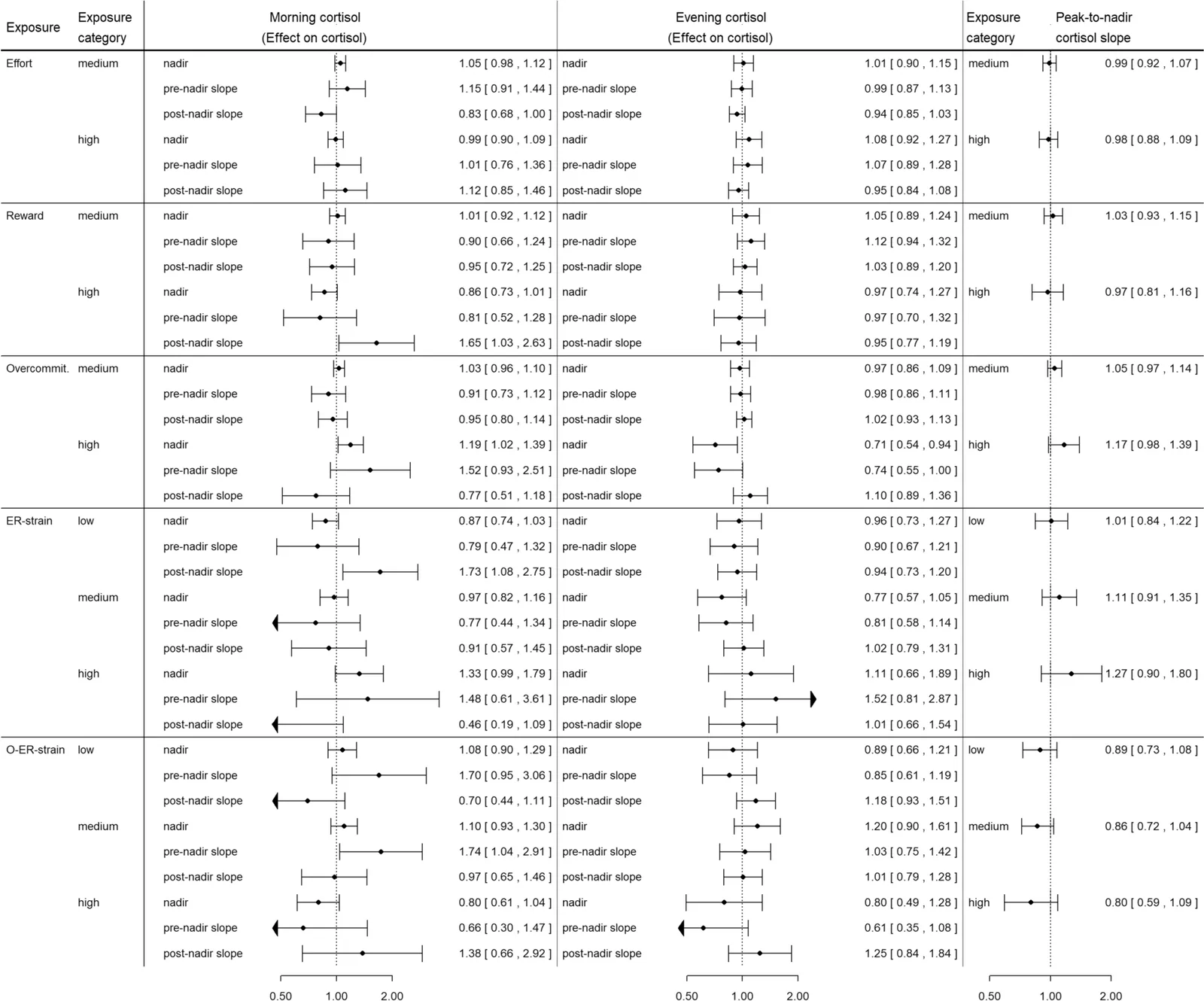
**Forest plots showing estimated effects of ERI categorical exposures on morning and evening cortisol patterns (peak or nadir concentrations with associated slopes) and on the morning-to-evening cortisol slope, mutually adjusted and adjusted for effects of potential confounders**. See explanatory notes in Legend to Figure 2. P-values for the null hypothesis that an exposure has no effect on the morning and evening cortisol pattern (peak or nadir concentrations with associated slopes, considered together) or on the morning-to-evening slope: *Morning cortisol pattern*: effort p=0.30; reward p=0.19; overcommitment p=0.17; ER-strain p=0.14; O-ER-strain p=0.18. *Evening cortisol pattern*: effort p=0.67; reward p=0.48; overcommitment p=0.29; ER-strain p=0.31; O-ER-strain p=0.29. *Morning-to-evening slope*: effort p=0.92; reward p=0.70; overcommitment p=0.19; ER-strain p=0.52; O-ER-strain p=0.19. Number of observations used in the analyses out of all observations with valid information on cortisol concentrations and saliva sampling times: morning cortisol n=5078/5978; evening cortisol n=5285/6230; morning-to-evening slope n=4941/5811.

The estimated effects of categorized DCS- and ERI-exposures are shown in Figs. 2 and 3.

The overall mutually adjusted effects of exposures on morning cortisol (peak concentrations and pre- and post-peak slopes considered together), evening cortisol (nadir concentrations and pre- and post-nadir slopes considered together) and on peak-to-nadir cortisol slopes (low, medium and high exposures considered together) was only statistically significant for two out of thirty categorical exposure-outcome relations (demands on evening cortisol (*p* = 0.02)) and support on peak-to-nadir slope (*p* = 0.04)) but not with a pattern consistent with allostatic overload (Fig. 2). Parallel analyses of continuous exposure effects showed no significant associations with cortisol outcomes (Supplementary material S7 (Model 3)).

### Effects of potential confounders on morning and evening cortisol.

**Fig. 4:**
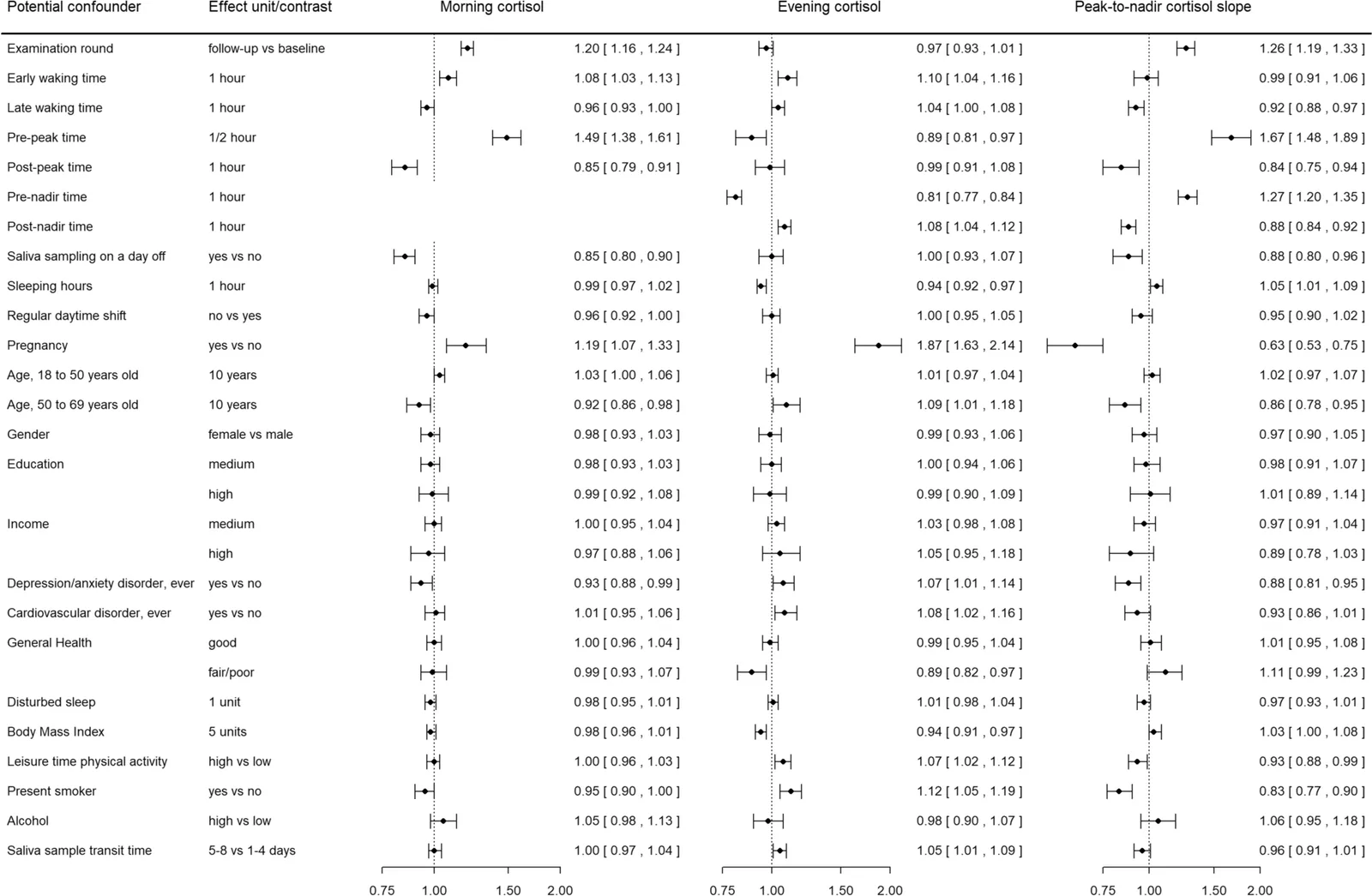
**Forest plots showing mutually adjusted effect estimates for potential confounders on morning and evening cortisol concentrations and on the morning-to-evening cortisol slope.** Estimated effects on morning and evening cortisol are the ratios by which the geometric mean cortisol concentration changes with a one unit change of continuous confounders or with respect to the reference category of categorical cofounders. Estimated effects on the morning-to-evening cortisol slope measure how the morning-to-evening ratio in the geometric mean cortisol concentrations changes with a one unit change of continuous confounders or with respect to the reference category of categorical cofounders. An effect ratio above unity implies that the morning-to-evening slope becomes steeper. P-values for the null hypothesis that a confounder with more than one associated parameter (time splines, age or non-binary categorical confounders) has no effect on the cortisol concentration: *Waking time*: morning cortisol **p=0.026**; evening cortisol **p=0.001**; morning-to-evening slope **p=0.004**. *Peak time*: morning cortisol **p=<0.0001**; evening cortisol **p=0.02**; morning-to-evening slope **p=<0.0001**. *Nadir time*: morning cortisol not applicable; evening cortisol **p=<0.001**; morning-to-evening slope **p=<0.0001**. *Age*: morning cortisol **p=0.03**; evening cortisol **p=0.03**; morning-to-evening slope **p=0.01**. *Education*: morning cortisol p=0.63; evening cortisol p=0.98; morning-to-evening slope p=0.84. *Income*: morning cortisol p=0.76; evening cortisol p=0.51; morning-to-evening slope p=0.29. *General health*: morning cortisol p=0.98; evening cortisol **p=0.02**; morning-to-evening slope p=0.17. Number of observations used in the analyses out of all observations with valid information on cortisol concentrations and saliva sampling times: morning cortisol n= 5174/5978; evening cortisol n=5387/6230; morning-to-evening slope n=5045/5811.

The mutually adjusted relations between potential confounders and morning and evening cortisol and the peak-to-nadir slope are shown in Fig. 4, ordered by sampling circumstances, age, gender, socioeconomic factors, health, and lifestyle factors. In contrast to the results for exposures, there were many significant associations (33/75).

There were highly significant effects of pre- and post-peak and -nadir times and of pregnancy, no effects of gender, education, income, disturbed sleep, and alcohol consumption. Other confounders were significantly associated with at least one cortisol outcome. Several associations were only borderline significant.

### Sensitivity analyses

The estimated effects of DCS- and ERI-exposures on cortisol were very similar in analyses with minimal, limited and full adjustment for potential confounders (Supplementary material S7). Analyses of models with only main exposures or only one of the composite variables did not indicate any attenuation bias caused by collinearity in the main analyses (Supplementary material S8).

## Discussion

We found no support for the hypothesis that DCS- or ERI-exposures are associated with changes of the diurnal cortisol concentrations in saliva samples.

Our study differs from most other studies on DCS- and ERI-exposure effects on diurnal cortisol by examining the independent effects of all DCS and ERI hypotheses in the same statistical model. The large majority of studies examine only effects of sub-parts of the stress models, typically effects of job-strain (Alves et al. 2013) or the ER-ratio (Lang et al. 2022) without considering how these effects are confounded by effects of the other parts of the stress models. This approach is in accordance with the statistical principle that inclusion of higher-order terms as explaining variables should be accompanied by inclusion of the related lower-order terms to avoid false positive and negative inferences (Brambor et al. 2006; Kronmal 1993; Lang et al. 2022), but some researchers disagree with this principle in relation to DCS and ERI composite variables (Dragano et al. 2018).

Another major difference is that we categorized continuous exposures by their verbal anchors in the questionnaires and not by percentile distributions. This approach allows a transparent and meaningful interpretation of category effects in relation to allostatic overload. Except for demands and effort, it revealed a low prevalence of DCS-exposures experienced ‘often’ or ‘always’ and ERI exposures experienced as bothering ‘somewhat’ or a ‘lot’, especially for composite variables (Tables 2 and 3). The implication is that only very large studies or studies of very poor work environments will have the power to detect if DCS and ERI exposures have an impact on diurnal cortisol concentrations consistent with the allostatic overload concept. Even in a study as large as ours, some confidence limits for high and medium levels of interaction terms were wide (Figs. 2 and 3), and the study was not large enough to study the separate effects of the two highest exposure categories.

### Exposures

Our DCS- and ERI-exposures were based on well documented methods. Their means and SD’s (considering the direction of control, support and rewards) were compatible with other studies using the same five-point scale scores across many different occupational groups, (DCS-exposures: Baka et al. 2022; Berthelsen et al. 2018; NFA 2007; Osman et al. 2021; Peter et al. 2022); ERI-exposures: (Herr et al. 2017b; Marchand et al. 2016; Planert et al. 2023; Siegrist et al. 2004; Violanti et al. 2018b; Wahrendorf et al. 2012). A recent meta-analysis of the prevalence of an ER-ratio>1 among physicians, using the same response options as in our study, found a meta-analytic prevalence of 21.1% (95% CI 16.3–21.7) based on 25 prevalence estimates (Le Huu et al. 2022). The corresponding baseline median prevalence of an ER-ratio > 1 in eleven European cohort studies was 16% (range 8–56%) (Dragano et al. 2017). Our ER-ratio > 1 frequencies were 27% at baseline and 22% at follow-up.

Exposure main scale internal item correlations (Cronbach’s alpha) and scale intercorrelations were also similar to those of other studies (Liao et al. 2013; Maina et al. 2009; Marchand et al. 2016; Montano et al. 2016; Preckel et al. 2007; van Vegchel et al. 2005). Thus, exposure levels and other properties of our DCS- and ERI-exposures are not very different from those of many other studies.

We found high correlations between composite exposure variables and their constituents. This is expected when one variable becomes part of another. Similar high correlations have been found in other studies (Liao et al. 2013; Maina et al. 2009; Marchand et al. 2016; Montano et al. 2016; Preckel et al. 2007; van Vegchel et al. 2005; Violanti et al. 2018a). However, sensitivity analyses did not indicate that the main analyses missed any important information due to attenuation caused by collinearity between exposure variables

### Cortisol and sampling times

Cortisol analyses were made with an established and well documented method (Hansen et al. 2003), and information on waking time and sampling times recorded in the sampling log and on sample tube labels had to be consistent and within specified time criteria. Cortisol and sampling time data validated by these criteria comprised approximately 5000 data points for analyses of morning and evening cortisol and peak-to-nadir cortisol slopes.

It is a limitation that we had only cortisol measurements on a single day, that saliva sampling times were self-reported, and that pre-sampling behavioral restrictions (eating, drinking, smoking, physical activity) were not requested. If errors related to these limitations are independent of exposures and true confounders they will reduce the power of the study, but effect estimates will not be biased. In this situation, a large study sample will counteract effects of error. Furthermore, it seems unlikely that error in cortisol concentrations and saliva sampling times would specifically bias results for true effects of DCS- and ERI-exposures towards the null to an extent of finding no associations, and at the same time allow demonstration of many significant associations with potential confounders, including those with established and consistent associations such as levels and slopes of the normal circadian cortisol pattern, taking saliva samples on a day off versus on a work day, pregnancy, and perhaps effects of older age on evening concentrations and the diurnal slope (Karlamangla et al. 2022; Marchand et al. 2016; Miller et al. 2016; Stalder et al. 2016).

Unexpectedly, morning cortisol increased significantly from baseline to follow-up. Saliva sampling methods, instructions, sampling times, laboratory and laboratory methods were the same across examination rounds. Selection, changes in cortisol determinants and a shorter storage time at follow-up may explain some of the increase (Mikkelsen et al. 2017). A further explanation may be a cotton tampon batch change. Cortisol associations to awakening and sampling times and to other determinants of cortisol, including DCS- and ERI-exposures, were similar at baseline and at follow-up. We therefore consider the drift in morning cortisol concentrations from baseline to follow-up as non-differential in relation to DCS- and ERI-exposures. In the analyses, we adjusted for a temporal drift by controlling for effects of examination round.

### Bias and confounding

Cross-sectional and two-year longitudinal effects of DCS- and ERI-exposures and of potential confounders were similar, indicating that unmeasured intraindividual factors were unlikely to confound cross-sectional effects in this material with two years of follow-up (Supplementary material S3).

We used a comprehensive set of potential confounders. In contrast to findings for DCS- and ERI exposures, there were many significant associations between potential confounders and measures of diurnal cortisol. However, they hardly acted as true confounders since the relations of DCS- and ERI-exposures with cortisol outcomes were very similar in analyses adjusting for all, a limited number, or no potential confounders

The literature on associations between potential confounders included in this study and diurnal cortisol concentrations is generally rather inconsistent. However, there is general agreement about the basic diurnal cortisol pattern in healthy adults (Karlamangla et al. 2013; Miller et al. 2016; Stalder et al. 2016). Furthermore, the evidence that morning cortisol is lower on a day off compared to work-days seems quite consistent (Bellingrath et al. 2008; Karlamangla et al. 2013; Kunz-Ebrecht et al. 2004; Marchand et al. 2013). This also applies to pregnancy associations with higher diurnal cortisol levels and a flatter peak-to-nadir slope (Jung et al. 2011; Obel et al. 2005; Pivonello et al. 2025; Schowe et al. 2024). Several studies also indicate that older persons have higher evening concentrations and flatter morning-to-evening slopes than younger persons (Adam et al. 2006; Dmitrieva et al. 2013; Karlamangla et al. 2013; Larsson et al. 2009; Nater et al. 2013). Our results are consistent with these findings. Other findings are supported by some studies and not by other studies. A qualified opinion on consistency or not for these findings would require systematic reviews applicable to characteristics of our study population. This is beyond the scope of this study.

Characteristics of participants who participated in both examination rounds compared to those who only participated in the first round may indicate selection related to SES factors (Table 4). However, these factors did not confound the relation between exposures and cortisol. Furthermore, selection was not related to exposure per se. Altogether, it seems unlikely that selection bias would have a significant influence on our results.

### Statistical models

We analysed the diurnal log-transformed cortisol concentrations as outcome with linear splines to model the effect of sampling times morning and evening. This method has previously been shown to comply well with observed data for daytime trajectories of cortisol and with more complicated statistical models (Karlamangla et al. 2013; Ranjit et al. 2005).

### Other studies

We are aware of only two other studies that examined effects of mutually adjusted DCS or ERI exposures on saliva cortisol in the same statistical model (Gadinger et al. 2011; Marchand et al. 2016). They found no significant interaction effects and only one significant main effect (job demands squared, 0.01 < *p* < 0.05 (Gadinger et al. 2011).

One systematic review on associations of circadian cortisol characteristics with mainly DCS- and ERI-exposures (Karlson et al. 2012) and one with meta-analyses on associations between the CAR and job stress in general (Boggero et al. 2017) found no consistent evidence of significant associations. Karlson et al. (2012) concluded that relatively few significant findings could be explained by rather small study groups and fairly homogeneous psychosocial work stressors of only mild or moderate intensity, and that the results indicate a normal, healthy response to work stress in most workers. Boggero et al. (2017) concluded that true associations could neither be excluded nor supported with confidence.

A third systematic review (Siegrist and Li 2016) on the relation of main effects of overcommitment and interactions of overcommitment with the ER-ratio on various cortisol outcomes concluded that findings were ‘inconsistent’. A fourth systematic review with meta-analyses (Eddy et al. 2023) examined effects of the ER-ratio and overcommitment on various categories of cortisol concentrations in saliva. The ER-ratio was positively associated with increasing waking cortisol in meta-analysis of six estimates (*p* = 0.02), and overcommitment was negatively associated with cortisol in the afternoon in meta-analysis of two estimates (*p* = 0.02).

The median number of participants per study in these reviews varied from 70 to 115, and only six studies included more than 300 participants. Power calculations by Boggero et al. (2017) showed that cross-sectional studies of CAR-outcomes would need a minimum sample size of more than 500 persons to demonstrate a true effect with a correlation set to 0.10 with 80% power.

Hair cortisol concentrations (HCC) is an alternative way of assessing relations between exposure to stressors and cumulated cortisol secretion during a specified time period (1–3 months). However, changes in diurnal levels and slopes are lost. A systematic review with meta-analyses of associations between HCC and chronic stress-exposure showed a positive association with ongoing but not with past chronic exposure, and not with perceived stress (Stalder et al. 2017).

Studies on DCS- and ERI-exposure effects on HCC are few, indicating no associations for DCS-exposures (Gidlow et al. 2016; Herr et al. 2017a), and inconsistent associations for ERI-exposures (Janssens et al. 2017; Qi et al. 2014, 2015).

### Strengths and limitations

The main strength of this study is the large study sample in combination with analyses of mutually adjusted effects of all exposure components of the DCS and ERI stress hypotheses. It is also a strength that DCS- and ERI-exposure categories refer to verbal anchors of questionnaire items, allowing transparent interpretations that fit to the allostatic overload theory. Furthermore, a large proportion of exposure-outcome data points (42%) were prospective across two calendar years.

Main limitations are morning and evening cortisol patterns being assessed from inter-individual cortisol data, that saliva samples were only collected for one day, that sampling times were self-reported, that pre-sampling behavioral restrictions were not requested, and that non-participation was high at follow-up. However, these limitations are hardly differential in relation to the associations between the diurnal cortisol pattern and DCS- and ERI-exposures

### Perspectives and suggestions

The results of this study are intriguing. If DCS- and ERI-exposures do not impact the function of the HPA-axis system in accordance with the allostatic overload theory, how likely is it then that they may do so for other allostatic physiological systems? And if unlikely, what are the explanations then for associations of DCS- and ERI-exposures with medical disorders and other measures of ill health?

We suggest that future DCS and ERI studies declare exposure distributions and category cut points by original scale scores to improve understanding and comparison of results with other studies.

## Conclusion

In conclusion, our results do not support the hypothesis that exposure to work-related stressors specified in the DCS- and ERI-stress theories are associated with changes in the diurnal pattern of cortisol saliva concentrations in a large Danish population of municipality civil servants.

## Supplementary Information

Below is the link to the electronic supplementary material.

Supplementary file1 (DOCX 738 KB)

## Data Availability

Data will be made available on reasonable request in accordance with conditions set by the Danish Data, Protection Agency (2009–41-3215).
