# Supplementary material for: Associations between work stressors, measured by the demand-control-support and effort-reward-imbalance models, and diurnal cortisol concentrations in saliva
Source: Int Arch Occup Environ Health. 2026 Sep 9;99(7):50. doi: 10.1007/s00420-026-02209-3 (PMC13558388; doi:10.1007/s00420-026-02209-3)
Supplement: Supplementary file 1 [file 420_2026_2209_MOESM1_ESM.docx]

**Supplementary material**

Online supporting information for the following article published in *International Archives of Occupational and Environmental Health*

**Associations between work stressors, measured by the demand-control-support and effort-reward-imbalance models, and diurnal cortisol concentrations in saliva**

Sigurd Mikkelsen^1^, Julie Lyng Forman^2^, Johan Hviid Andersen^3^, Esben Meulengracht Flachs^1^, Matias Brødsgaard Grynderup^4,7^, Åse Marie Hansen^5,6^, Linda Kaerlev^4,8^, Henrik Albert Kolstad^4^, Reiner Rugulies^5,6^,Stinna Skaaby^1^, Jane Frølund Thomsen^1,^ Jens Peter Bonde.^1^

^1^ Department of Occupational and Environmental Medicine, Bispebjerg and Frederiksberg University Hospital, Copenhagen, Denmark.

^2^ Section of Biostatistics, Department of Public Health, University of Copenhagen, Copenhagen, Denmark.

^3^ Occupational and Environmental Medicine, Danish Ramazzini Centre, Goedstrup Hospital, Herning, Denmark.

^4^ Department of Occupational Medicine, Danish Ramazzini Centre, Aarhus University Hospital, Århus, Denmark.

^5^ Department of Public Health, University of Copenhagen, Denmark.

^6^ National Research Centre for the Working Environment, Copenhagen, Denmark.

^7^ Psychiatric Center North Zeeland, Copenhagen University Hospital, Capital Region of Denmark Mental Health, Services, Hillerød, Denmark.

^8^Research Unit of Clinical Epidemiology, Institute of Clinical Research, University of Southern Denmark, Odense, Denmark.

**Content**

S1. DCS and ERI questionnaire items, response options and scale score calculations.	p3

S2. Statistical models of exposure effects on the diurnal cortisol pattern.	p5

S3. Cross-sectional and longitudinal exposure effects on morning and evening cortisol concentrations.	p7

S4. Table 1 in the main text, by examination round.	p12

S5. Correlations between DCS-exposure scales and between ERI-exposure scales.	p13

S6. Four-level categorical job-strain and ER-strain exposure variables.	p14

S7. Influence of level of confounder control on effects of DCS- and ERI-exposures	p15

S8. Sensitivity analyses of possible effect attenuation due to collinearity between exposure variables.	p20

**S1. DCS and ERI questionnaire items, response options and scale score calculations**

**Textbox S1_1**. Items measuring the level of quantitative psychological demands, control (subscales skill discretion and decision authority) and support at work. Left column shows the items numbered by their order in the questionnaire.

| **Item #** | **Quantitative psychological demands** |
| --- | --- |
| 1 | Is your workload unevenly distributed so it piles up? |
| 2 | How often do you not have time to complete all your work tasks? |
| 3 | Do you get behind with your work? |
| 4 | Do you have enough time for your work tasks? |
|  | **Skill discretion** |
| 5 | Does your work require you to take the initiative? |
| 6 | Do you have the possibility of learning new things through your work? |
| 7 | Can you use your skills or expertise in your work? |
| 8 | Does your work give you the opportunity to develop your skills? |
|  | **Decision authority** |
| 9 | Do you have a large degree of influence concerning your work? |
| 10 | Do you have a say in choosing who you work with? |
| 11 | Can you influence the amount of work assigned to you? |
| 12 | Do you have any influence on what you do at work? |
|  | **Support** |
| 13 | If you have problems at work, can you get the necessary help and support from your superiors? |
| 14 | If you have problems at work, can you get the necessary help and support from your colleagues? |

Response options for items #1 to #12 (scores): ‘always’ (1), ‘often’ (2), ‘sometimes’ (3), ‘seldom’ (4), ‘never/almost never’ (5).

Response options for items #13 and #14 (scores): ‘always’ (1), almost always’ (2), ‘often’ (3), ‘sometimes’ (4), ‘seldom/never’ (5).

Scale scores for each construct were calculated as the mean of item scores. Demand items #1 to #3 were reversed before calculating the mean. Scale scores for control were calculated as the mean of the scale scores of skill discretion and decision authority. Scale scores were ordered to increase with increasing demands, decreasing control, and decreasing support.

Considering that three of four demand item scores were reversed to express increasing demands, the descriptive labels must also be reversed. However, reversed labels are not identical to the labels of the non-reversed item score, although the meaning is very much the same. We will interpret the verbal anchors of the increasing demand scale score as 1=’never/almost never’, 2=’seldom’, 3=’sometimes’, 4=’often’ and 5=’always’.

**Textbox S1_2.** Statement items measuring the level of effort, reward and overcommitment. Left column shows the items numbered by their order in the questionnaire.

| **Item #** | **Effort** |
| --- | --- |
| 1 | I have constant time pressure due to a heavy workload |
| 2 | I have many interruptions and disturbances in my job |
| 3 | Over the past few years, my job has become more and more demanding |
|  | **Reward** |
| 4 | I have experienced or I expect to experience an  undesirable change in my work situation |
| 5 | My job security is poor |
| 6 | My job promotion prospects are poor |
| 7 | I receive the respect I deserve from my superiors |
| 8 | Considering all my efforts and achievements, I receive the respect and prestige I deserve at work |
| 9 | Considering all my efforts and achievements,  my work prospects are adequate |
| 10 | Considering all my efforts and achievements, my salary/income is adequate |
|  | **Overcommitment** |
| 11 | I get easily overwhelmed by time pressures at work |
| 12 | As soon as I get up in the morning, I start thinking about work problems |
| 13 | When I get home, I can easily relax and ‘switch off’ work |
| 14 | People close to me say I sacrifice too much for my job |
| 15 | Work rarely lets me go, it is still on my mind when I go to bed |
| 16 | If I postpone something that I was supposed to do today, I’ll have trouble sleeping at night |

The ERI item statements were presented after an initial statement ‘How do the following statements apply for you?’

Response options, scoring and calculation of scale scores, see text. Item scores for item #13 were reversed in calculations of the overcommitment scale score.

Effort and reward items were scored by the original two-step response option format. The first step was to agree or disagree with the item statement. If agreeing to the statement, the next step was to state the degree to which the respondent was bothered by the presence of this aspect. Response option scores for items #1-#6 were: 1= ‘disagree with statement’, 2=’agree, it does not bother me’. 3=’agree, it bothers me a little, 4=agree, it bothers me somewhat, 5=agree, it bothers me a lot’. Response option scores for items #7-#10 were: 1= ‘agree with statement’, 2=’disagree, it does not bother me’. 3=’disagree, it bothers me a little, 4=disagree, it bothers me somewhat, 5=disagree, it bothers me a lot’.

Response options for overcommitment item statements were ‘strongly disagree’, ‘disagree’, ‘agree’, ‘strongly agree’, scored 1 to 4.

**S2. Statistical models of exposure effects on the diurnal cortisol pattern**

Fig. S2 shows a linear spline approximating the morning and evening pattern of ln(cortisol concentration) in saliva. The figure illustrates the effect parameters in the present study: the morning peak level, the pre-peak slope, the post-peak slope, the evening nadir level, the pre-nadir slope, the post-nadir slope, and the morning-to-evening slope which is represented by the yellow line from the peak to the nadir. Note that the sampling time covariate has been defined with peak- and nadir-times as its reference point.

Fig. S2: Linear spline model for the morning and evening pattern of cortisol concentrations in saliva.

**Analytic models**

Notation:

DCS exposures: E1=demands, E2=control, E3=support, E4=job-strain, E5=iso-strain.

ERI exposures: E1=effort, E2=reward, E3=overcommitment. E4=ER-ratio and E5=O-ER-ratio for continuous exposures. E4=ER-strain and E5=O-ER-strain for categorical exposures.

α is the intercept, β’s are regression coefficients, ε is random error.

***Morning cortisol***

1. The linear mixed model for *continuous* exposures is specified by:

ln(morning cortisol) = α + β_1_*pre-peak-time + β_2_*post-peak-time +

β_E1_*E1 + β _E11_*E1*pre-peak-time + β _E12_*E1*post-peak-time +

β_E2_*E2 + β _E21_*E2*pre-peak-time + β _E22_*E2*post-peak-time +

β_E3_*E3 + β _E31_*E3*pre-peak-time + β _E32_*E3*post-peak-time +

β_E4_*E4 + β _E41_*E4*pre-peak-time + β _E42_*E4*post-peak-time +

β_E5_*E5 + β _E51_*E5*pre-peak-time + β _E52_*E5*post-peak-time +

[effects of potential confounders] +

[random effect of study participant] + ε

Potential confounders included all potential confounders except evening saliva sampling times.

2. The linear mixed model for *categorical* exposures included categories of E1 to E5 as dummy variables with reference to the lowest exposure group together with their multiplicative interactions with pre- and post-peak-times.

Estimated exposure effects were reported as back-transformed regression coefficients, thus becoming ratio changes in geometric mean of cortisol peak concentration and ratio changes in rate of incline or decline in associated slopes.

***Evening cortisol***

Similar models were used to analyze evening cortisol except that pre- and post-peak times were replaced with pre- and post-nadir times. Potential confounders included all potential confounders. Early and late waking time-splines and pre- and post-peak time-splines were included because they may affect evening cortisol.

***Morning-to-evening cortisol slope***

1. The linear mixed model for continuous exposures was specified by:

(ln(morning cortisol) – ln(evening cortisol)=

α + β_1_*pre-peak-time + β_2_*post-peak-time + β_3_*pre-nadir-time + β_4_*post-nadir-time +

β_E1_*E1 + β_E2_*E2 + β_E3_*E3 + β_E4_*E4 + β_E5_*E5 +[effects of potential confounders] +

[random effect of study participant] + ε

Potential confounders included all potential confounders, they may affect the morning-to-evening slope.

2. The linear mixed model for categorical exposures included exposure categories E1 to E5 as dummy variables with reference to the lowest exposure group.

Estimated exposure effects were presented as back-transformed regression coefficients (β_E1_ to β_E5_), thus becoming ratio changes of the ratio between geometric mean cortisol concentration at peak (nominator) and at nadir (denominator).

**NOTE:** In the analyses of morning and evening cortisol, the effects of exposures on the pre- and post- peak/nadir slopes are assessed from the interaction terms between the exposures and the pre- and post- spline-time components, while adjusting for the main effects of the exposures and the sampling spline-time. This implies that the estimated main effects of the exposures refer to the reference-point of the sampling time covariate which we set to the peak time and nadir time in our main analyses.

Exposure effects on waking concentrations may also be of interest and we therefore made supplementary analyses of morning cortisol with the reference time set to the waking time. DCS- and ERI-exposures had no effect on waking time cortisol. However, the confidence limits were quite wide (data not shown).

Since exposures are not included in any interaction terms in analyses of the morning-to-evening slopes, exposure effects on the waking-to-nadir slope are the same as on the peak-to-nadir slopes. However, this measure includes the CAR, which is excluded in most other assessments of the waking-to-nadir slopes.

**S3. Cross-sectional and longitudinal exposure effects on morning and evening cortisol**

As stated in the main text, we tested if cross-sectional and longitudinal effects on morning and evening cortisol concentrations were different for exposures and time-varying potential confounders, using the method described by Fitzmaurice et al. (2011) (see reference in the main text). This method is also referred to as the *Hausman test for unmeasured confounders* because differences between the cross-sectional and longitudinal effects may indicate a bias in the cross-sectional effect due to uncontrolled confounding.

The results are shown below (Tables S3_1 to S3_3). Hausman tests were not applicable (n.a.) if cross-sectional and longitudinal variables were highly correlated. Out of 68 Hausman tests, only three revealed significant differences (p=0.02, 0.03 and 0.04). Overall, there were no systematic differences between cross-sectional and longitudinal effects of exposures or potential confounders.

**Table S3_1. Cross-sectional and longitudinal effects of DCS-exposures on geometric mean cortisol concentrations (nmol/l), mutually adjusted and adjusted for effects of potential confounders.**

| **DCS** | | **Expo-sure type**^d^ | **Morning cortisol**^a^  (n=5105/5978)^c^ | | | | **Evening cortisol**^b^  ((n=5317/6230)^c^ | | | |
| --- | --- | --- | --- | --- | --- | --- | --- | --- | --- | --- |
| **Continuous**  **exposures** | |  | **Effect ratio**^e^ | **95% CI** | **p**^f^ | **Hausman test p**^g^ | **Effect ratio**^e^ | **95% CI** | **p**^f^ | **Hausman test p**^g^ |
| Demands | | cross | 1.03 | 0.91 -1.17 | 0.62 | 0.56 | 0.97 | 0.84 -1.12 | 0.67 | 0.72 |
|  |  | long | 0.97 | 0.81 -1.16 | 0.73 |  | 1.02 | 0.81 -1.28 | 0.88 |  |
| Control | | cross | 1.05 | 0.92 -1.21 | 0.46 | 0.48 | 0.90 | 0.76 -1.06 | 0.19 | 0.18 |
|  |  | long | 0.97 | 0.79 -1.19 | 0.75 |  | 1.10 | 0.85 -1.41 | 0.47 |  |
| Support | | cross | 1.01 | 0.94 -1.08 | 0.81 | 0.60 | 1.05 | 0.97 -1.13 | 0.23 | 0.31 |
|  |  | long | 1.04 | 0.95 -1.14 | 0.42 |  | 0.98 | 0.87 -1.10 | 0.70 |  |
| Job-strain | | cross | 0.94 | 0.70 -1.25 | 0.65 | 0.51 | 1.19 | 0.84 -1.68 | 0.33 | 0.47 |
|  |  | long | 1.10 | 0.73 -1.65 | 0.65 |  | 0.96 | 0.58 -1.58 | 0.86 |  |
| Iso-strain | | cross | 1.03 | 0.84 -1.27 | 0.78 | 0.64 | 0.85 | 0.67 -1.09 | 0.20 | 0.32 |
|  |  | long | 0.95 | 0.71 -1.26 | 0.72 |  | 1.05 | 0.74 -1.50 | 0.77 |  |
| **Categorical exposures** | **Catego-ries^h^** | **Expo-sure type**^d^ | **Effect ratio**^e^ | **95% CI** | **p**^f^ | **Hausman test p**^g^ | **Effect ratio**^e^ | **95% CI** | **p**^f^ | **Hausman test p**^g^ |
| Demands | low |  | 1 |  |  |  | 1 |  |  |  |
|  | medium | cross | 0.99 | 0.92 -1.07 | 0.79 | 0.55 | 1.01 | 0.93 -1.11 | 0.81 | 0.93 |
|  |  | long | 1.03 | 0.93 -1.13 | 0.59 |  | 1.00 | 0.89 -1.13 | 0.94 |  |
|  | high | cross | 1.01 | 0.93 -1.10 | 0.76 | 0.92 | 1.10 | 1.00 -1.21 | 0.06 | 0.29 |
|  |  | long | 1.01 | 0.89 -1.13 | 0.92 |  | 1.00 | 0.87 -1.16 | 0.97 |  |
| Control | high |  | 1 |  |  |  | 1 |  |  |  |
|  | medium | cross | 1.02 | 0.94 -1.10 | 0.68 | 0.39 | 0.98 | 0.89 -1.08 | 0.76 | 0.72 |
|  |  | long | 1.07 | 0.97 -1.18 | 0.17 |  | 0.96 | 0.85 -1.09 | 0.50 |  |
|  | low | comb | 1.01 | 0.89 -1.14 | 0.91 | n.a. | 0.98 | 0.85 -1.13 | 0.77 | n.a. |
| Support | high |  | 1 |  |  |  | 1 |  |  |  |
|  | medium | cross | 1.03 | 0.94 -1.13 | 0.52 | 0.09 | 1.10 | 0.99 -1.22 | 0.09 | 0.98 |
|  |  | long | 0.94 | 0.85 -1.04 | 0.20 |  | 1.09 | 0.97 -1.24 | 0.15 |  |
|  | low | comb | 1.02 | 0.89 -1.16 | 0.76 | n.a. | 0.89 | 0.76 -1.04 | 0.13 | n.a. |
| Job-strain | very low |  | 1 |  |  |  | 1 |  |  |  |
|  | low | cross | 1.00 | 0.89 -1.12 | 0.97 | 0.60 | 1.09 | 0.95 -1.24 | 0.20 | 0.77 |
|  |  | long | 0.96 | 0.84 -1.09 | 0.49 |  | 1.06 | 0.90 -1.24 | 0.50 |  |
|  | medium | cross | 1.00 | 0.89 -1.13 | 0.94 | 0.70 | 0.90 | 0.79 -1.04 | 0.15 | 0.08 |
|  |  | long | 0.97 | 0.85 -1.12 | 0.70 |  | 1.07 | 0.91 -1.27 | 0.41 |  |
|  | high | comb | 0.89 | 0.70 -1.14 | 0.37 | n.a. | 0.90 | 0.67 -1.22 | 0.50 | n.a. |
| Iso-strain | very low |  | 1 |  |  |  | 1 |  |  |  |
|  | low | comb | 1.00 | 0.88 -1.13 | 0.94 | n.a. | 0.87 | 0.75 -1.01 | 0.07 | n.a. |
|  | medium | comb | 1.05 | 0.93 -1.19 | 0.40 | n.a. | 0.96 | 0.83 -1.11 | 0.56 | n.a. |
|  | high | comb | 1.19 | 0.88 -1.62 | 0.25 | n.a. | 1.00 | 0.69-1.45 | 0.99 | n.a. |

^a^ Exposure effects adjusted for early- and late waking times, pre- and post-peak time-splines, and all other potential confounders (excluding pre- and post-nadir time-splines).

^b^ Exposure effects adjusted for early- and late waking times, pre- and post-peak time-splines, pre- and post-nadir time-splines, and all other potential confounders.

^c^ Number of observations used in the analyses out of all observations with valid information on cortisol concentrations and relevant saliva sampling time information

^d^ ‘cross’: cross-sectional, ‘long’: longitudinal, ‘comb’: cross-sectional and longitudinal combined to avoid collinearity when the distribution of a potential confounder was very similar at baseline and follow-up.

^e^Effect ratios are the ratios by which the morning or evening geometric mean cortisol concentrations (nmol/l saliva) change with a one unit change of exposure or for a category level compared to the reference category.

^f^ p1: probability that the observed effect ratio differs from unity (test with one degree of freedom).

^g^ Hausman test p: probability that there is no difference between cross-sectional and longitudinal exposure effects on cortisol concentrations. Degrees of freedom is the number of effect estimates. The Hausman test is not applicable (n.a.) when cross_sectional and longitudinal exposures were combined (effect type=’comb’).

^h^ For definitions, see Table 2 and Table 3 in the main text. **Table S3_2. Cross-sectional and longitudinal effects of ERI-exposures on geometric mean cortisol concentrations (nmol/l), mutually adjusted and adjusted for effects of potential confounders.**

| **ERI** | | **Expo-sure type**^d^ | **Morning cortisol**^a^  (n=5078/5978)^c^ | | | | **Evening cortisol**^b^  (n=5285/6230)^c^ | | | |
| --- | --- | --- | --- | --- | --- | --- | --- | --- | --- | --- |
| **Continuous**  **exposures** | |  | **Effect ratio**^e^ | **95% CI** | **p**^f^ | **Hausman test p**^g^ | **Effect ratio**^e^ | **95% CI** | **p**^f^ | **Hausman test p**^g^ |
| Effort | | cross | 1.01 | 0.96 -1.06 | 0.72 | 0.78 | 0.99 | 0.93 -1.05 | 0.66 | 0.36 |
|  |  | long | 1.02 | 0.96 -1.09 | 0.53 |  | 1.03 | 0.95 -1.12 | 0.42 |  |
| 1/(6-reward) | | cross | 1.01 | 0.96 -1.07 | 0.65 | 0.95 | 0.97 | 0.91 -1.03 | 0.30 | 0.30 |
|  |  | long | 1.01 | 0.94 -1.08 | 0.78 |  | 1.02 | 0.94 -1.11 | 0.60 |  |
| Overcommitment | | cross | 1.00 | 0.92 -1.08 | 0.93 | 0,84 | 1.00 | 0.91 -1.10 | 0.96 | 0.25 |
|  |  | long | 0.98 | 0.88 -1.10 | 0.76 |  | 0.91 | 0.79 -1.05 | 0.19 |  |
| ER-ratio | | cross | 0.95 | 0.71 -1.27 | 0.73 | 0.95 | 1.03 | 0.73 -1.45 | 0.87 | 0.33 |
|  |  | long | 0.96 | 0.70 -1.33 | 0.83 |  | 0.80 | 0.53 -1.19 | 0.27 |  |
| O-ER-ratio | | cross | 1.04 | 0.77 -1.41 | 0.80 | 0.96 | 1.01 | 0.70 -1.45 | 0.96 | 0.41 |
|  |  | long | 1.05 | 0.72 -1.54 | 0.72 |  | 1.28 | 0.80 -2.03 | 0.30 |  |
| **Categorical exposures** | **Catego-ries^h^** | **Expo-sure type**^d^ | **Effect ratio^e^** | **95% CI** | **p^f^** | **Hausman test p**^g^ | **Effect ratio^e^** | **95% CI** | **p^f^** | **Hausman test p**^g^ |
| Effort | low |  | 1 |  |  |  | 1 |  |  |  |
|  | medium | cross | 0.96 | 0.90 -1.02 | 0.20 | **0.03** | 1.00 | 0.93 -1.08 | 0.96 | 0.91 |
|  |  | long | 1.07 | 0.99 -1.16 | 0.07 |  | 0.99 | 0.90 -1.10 | 0.91 |  |
|  | high | cross | 0.98 | 0.90 -1.06 | 0.59 | 0.25 | 1.01 | 0.92 -1.10 | 0.90 | 0.46 |
|  |  | long | 1.05 | 0.94 -1.14 | 0.39 |  | 1.06 | 0.93 -1.21 | 0.37 |  |
| Reward | high |  | 1 |  |  |  | 1 |  |  |  |
|  | medium | cross | 0.98 | 0.90 -1.07 | 0.64 | 0.13 | 0.95 | 0.85 -1.05 | 0.32 | 0-27 |
|  |  | long | 1.08 | 0.98 -1.19 | 0.13 |  | 1.03 | 0.91 -1.17 | 0.59 |  |
|  | low | comb | 0.96 | 0.85 -1.07 | 0.44 | n.a. | 0.99 | 0.86 -1.14 | 0.94 | n.a. |
| Overcommitment | low |  | 1 |  |  |  | 1 |  |  |  |
|  | medium | cross | 1.06 | 1.00 -1.12 | 0.07 | 0.37 | 1.01 | 0.94 -1.08 | 0.84 | 0.41 |
|  |  | long | 1.02 | 0.94 -1.09 | 0.69 |  | 0.96 | 0.88 -1.06 | 0.44 |  |
|  | high | comb | 1.08 | 0.97 -1.22 | 0.16 |  | 0.89 | 0.77 -1.02 | 0.09 | n.a. |
| ER-strain | very low |  | 1 |  |  |  | 1 |  |  |  |
|  | low | cross | 1.04 | 0.90 -1.21 | 0.59 | 0.13 | 1.02 | 0.86 -1.21 | 0.85 | 0.98 |
|  |  | long | 0.89 | 0.76 -1.05 | 0.17 |  | 1.01 | 0.83 -1.24 | 0.89 |  |
|  | medium | cross | 1.05 | 0.91 -1.21 | 0.52 | 0.14 | 0.92 | 0.78 -1.09 | 0.34 | 0.42 |
|  |  | long | 0.93 | 0.80 -1.09 | 0.36 |  | 0.85 | 0.70 -1.03 | 0.10 |  |
|  | high | comb | 1.12 | 0.90 -1.39 | 0.32 | n.a. | 0.86 | 0.66 -1.13 | 0.28 | n.a. |
| O-ER-strain | very low |  | 1 |  |  |  | 1 |  |  |  |
|  | low | comb | 0.95 | 0.84 -1.08 | 0.46 | n.a. | 1.05 | 0.90 -1.22 | 0.56 | n.a. |
|  | medium | comb | 1.01 | 0.90-1.14 | 0.89 | n.a. | 1.18 | 1.02 -1.37 | 0.02 | n.a. |
|  | high | comb | 0.89 | 0.73 -1.08 | 0.24 | n.a. | 1.12 | 0.89 -1.43 | 0.33 | n.a. |

^a-h^ See footnotes of Table S3_1

**Table S3_3. Cross-sectional and longitudinal effects of potential confounders on geometric mean cortisol concentrations (nmol/l), mutually adjusted.**

| **Potential confounder** |  |  | **Morning cortisol**  (n=5174/5978) ^a^ | | |  | **Evening cortisol**  (n=5387/6230) ^a^ | | |  |
| --- | --- | --- | --- | --- | --- | --- | --- | --- | --- | --- |
|  | **Covariate unit** | **Exposure type**^b^ | **Effect ratio^c^** | **95% CI** | **p^d^** | **Hausman test p**^e^ | **Effect ratio^c^** | **95% CI** | **p^d^** | **Hausman test p**^e^ |
| Examination round | follow-up vs baseline |  | 1.20 | 1.16 -1.24 | <0.0001 | n.a | 0.97 | 0.92 -1.01 | 0.16 | n.a |
| Early waking-time | per 1 hour | cross | 1.11 | 1.04 -1.17 | 0.0008 | 0.20 | 1.07 | 1.00 -1.15 | 0.04 | 0.32 |
|  |  | long | 1.03 | 0.94 -1.13 | 0.57 |  | 1.15 | 1.02 -1.30 | 0.02 |  |
| Late waking-time | per 1 hour | cross | 0.94 | 0.91 -0.98 | 0.001 | **0.02** | 1.06 | 1.02 -1.11 | 0.007 | 0.11 |
|  |  | long | 1.02 | 0.96 -1.08 | 0.58 |  | 0.99 | 0.93 -1.07 | 0.88 |  |
| Pre-peak time | per ½ hour | cross | 2.36 | 1.92 -2.90 | <0.0001 | 0.29 | 0.71 | 0.55 -0.91 | 0.006 | 0.22 |
|  |  | long | 2.01 | 1.61 -2.51 | <0.0001 |  | 0.89 | 0.68 -1.18 | 0.42 |  |
| Post-peak time | per 1 hour | cross | 0.82 | 0.75 -0.90 | <0.0001 | 0.32 | 0.97 | 0.87 -1.08 | 0.55 | 0.43 |
|  |  | long | 0.88 | 0.79 -0.9 | 0.02 |  | 1.04 | 0.91 -1.19 | 0.59 |  |
| Pre-nadir time | per 1 hour | cross | n.i.^f^ |  |  |  | 0.81 | 0.76 -0.86 | <0.0001 | 0.71 |
|  |  | long | n.i.^f^ |  |  |  | 0.80 | 0.74 -0.85 | <0.0001 |  |
| Post-peak time | per 1 hour | cross | n.i.^f^ |  |  |  | 1.08 | 1.03 -1.13 | 0.0008 | 0.89 |
|  |  | long | n.i.^f^ |  |  |  | 1.08 | 1.03 -1.14 | 0.002 |  |
| Saliva samples on day off | yes vs no | cross | 0.83 | 0.77 -0.90 | <0.0001 | 0.37 | 0.95 | 0.87 -1.04 | 0.25 | 0.07 |
|  |  | long | 0.88 | 0.80 -0.96 | 0.005 |  | 1.08 | 0.96 -1.21 | 0.19 |  |
| Sleeping hours | per 1 hour | cross | 1.00 | 0.97 -1.02 | 0.83 | 0.75 | 0.94 | 0.91 -0.97 | 0.0002 | 0.87 |
|  |  | long | 0.99 | 0.95 -1.03 | 0.60 |  | 0.95 | 0.90-1.00 | 0.03 |  |
| Regular daytime shift | no vs yes | comb | 0.96 | 0.92 -1.00 | 0.08 | n.a. | 1.00 | 0.95 -1.05 | 0.97 | n.a. |
| Pregnancy | yes vs no | comb | 1.18 | 1.06 -1.32 | 0.003 | n.a. | 1.85 | 1.62 -2.13 | <0.0001 | n.a. |
| Saliva sample delivery delay (days) | 5-8 vs 1-4 days | cross | 0.99 | 0.94 -1.04 | 0.63 | 0.50 | 1.05 | 0.99 -1.11 | 0.08 | 0.84 |
|  |  | long | 1.01 | 0.96 -1.06 | 0.64 |  | 1.04 | 0.98-1.11 | 0.17 |  |

(continues on next page)

**Table S3_3 (continued). Mutually adjusted cross-sectional and longitudinal effects of potential confounders on cortisol concentrations (nmol/l)**

| **Potential confounder** |  |  | **Morning**  (n=5174/5978) ^a^ | | |  | **Evening**  (n=5728/6640) ^a^ | | |  |
| --- | --- | --- | --- | --- | --- | --- | --- | --- | --- | --- |
|  | **Covariate unit** | **Exposure type**^b^ | **Effect ratio^c^** | **95% CI** | **p^d^** | **Hausman test p**^e^ | **Effect ratio^c^** | **95% CI** | **p^d^** | **Hausman test p**^e^ |
| Age, 18 to 50 years old | per 10 years | comb | 1.03 | 1.00 -1.06 | 0.10 | n.a. | 1.00 | 0.97 -1.04 | 0.84 | n.a. |
| Age, 50 to 69 years old | per 10 years |  | 0.93 | 0.87 -0.99 | 0.03 | n.a. | 1.10 | 1.01 -1.19 | 0.02 | n.a. |
| Gender | female vs male | comb | 0.98 | 0.94 -1.04 | 0.52 | n.a. | 1.00 | 0.90 -1.09 | 0.95 | n.a. |
| Education^a^ | low |  | 1 |  |  |  | 1 |  |  |  |
|  | medium | comb | 0.97 | 0.92 -1.03 | 0.31 | n.a. | 1.00 | 0.94 -1.07 | 0.97 | n.a. |
|  | high | comb | 0.99 | 0.91 -1.07 | 0.79 | n.a. | 0.99 | 0.90 -1.08 | 0.88 | n.a. |
| Income^a^ | low |  | 1 |  |  |  | 1 |  |  |  |
|  | medium | cross | 1.00 | 0.95 -1.05 | 0.95 | 0.39 | 1.04 | 0.98 -1.10 | 0.23 | 0.53 |
|  |  | long | 0.97 | 0.90 -1.04 | 0.34 |  | 1.00 | 0.92 -1.10 | 0.96 |  |
|  | high | comb | 0.96 | 0.87-1.05 | 0.37 | n.a. | 1.06 | 0.95 -1.18 | 0.29 | n.a. |
| Depression/anxiety disorder | yes vs no | comb | 0.94 | 0.89 -0.99 | 0.02 | n.a. | 1.08 | 1.01 -1.15 | 0.03 | n.a. |
| Cardiovascular disorder | yes vs no | comb | 1.01 | 0.95 -1.06 | 0.85 | n.a. | 1.08 | 1.02 -1.16 | 0.01 | n.a. |
| General health^a^ | excellent/very good |  | 1 |  |  |  | 1 |  |  |  |
|  | good | cross | 1.01 | 0.96 -1.06 | 0.77 | 0.80 | 1.00 | 0.94 -1.06 | 0.95 | 0.62 |
|  |  | long | 1.00 | 0.93 -1.07 | 0.93 |  | 0.97 | 0.90 -1.06 | 0.53 |  |
|  | fair/poor | comb | 1.00 | 0.93 -1.07 | 0.98 | n.a. | 0.89 | 0.82 -0.96 | 0.004 | n.a. |
| Disturbed sleep  (scale scores 1-5) | per unit | cross | 0.97 | 0.94 -1.00 | 0.05 | 0.15 | 1.00 | 0.97 -1.04 | 0.86 | 0.53 |
|  |  | long | 1.02 | 0.96 -1.07 | 0.58 |  | 1.03 | 0.96 -1.10 | 0.42 |  |
| Body mass index | per 5 units | comb | 0.99 | 0.96 -1.01 | 0.28 | n.a. | 0.94 | 0.91 -0.97 | <0.0001 | n.a. |
| Leisure time physical activity | high vs low | cross | 1.00 | 0.96 -1.04 | 0.99 | 0.85 | 1.04 | 0.98 -1.09 | 0.19 | **0.04** |
|  |  | long | 0.99 | 0.93 -1.06 | 0.82 |  | 1.15 | 1.06 -1.25 | 0.001 |  |
| Present smoker | yes vs no | comb | 0.95 | 0.90 -1.00 | 0.07 | n.a. | 1.11 | 1.03 -1.16 | 0.001 | n.a. |
| Alcohol consumption | high vs low | comb | 1.05 | 0.98 -1.13 | 0.15 | n.a. | 0.99 | 0.91 -1.07 | 0.75 | n.a. |

^a^ Number of observations used in the analyses out of all observations with valid information on cortisol concentrations and relevant saliva sampling time information

^b^ ‘cross’: cross-sectional, ‘long’: longitudinal, ‘comb’: cross-sectional and longitudinal combined to avoid collinearity when the distribution of a potential confounder was very similar at baseline and follow-up.

^c^Effect ratios are the ratios by which the morning or evening geometric mean cortisol concentrations (nmol/l saliva) change with a one unit change of exposure or for an exposure category compared to the reference category.

^d^ p: probability that the observed effect ratio differs from unity (test with one degree of freedom).

^e^ Hausman test p: probability that there is no difference between cross-sectional and longitudinal exposure effects on cortisol concentrations. Degrees of freedom is the number effect estimates. The Hausman test is not applicable (n.a.) when cross_sectional and longitudinal exposures were combined (effect type=’comb’).

^f^ n.i.: not included as potential confounder.

**S4 Table 1 in the main text, by examination round**

**Table S4 Distribution of number of valid cortisol saliva samples (n) by saliva sampling times with associated geometric means and 95% confidence intervals (CI) of cortisol concentrations (nmol/l). By examination round.**

| **Saliva sampling times** | **Baseline** | | |  | **Follow-up** | | |
| --- | --- | --- | --- | --- | --- | --- | --- |
| **Morning** | n | Cortisol (nmol/l) | |  | n | Cortisol (nmol/l) | |
| Minutes from waking |  | geometric mean | 95% CI |  |  | geometric mean | 95% CI |
| 0 - <5 | 93 | 7.6 | 6.4 -9.1 |  | 88 | 9.0 | 7.8 -10.4 |
| 5 - <15 | 82 | 8.1 | 6.8 -9.6 |  | 60 | 9.5 | 7.8 -11.5 |
| 15 - <20 | 162 | 8.9 | 8.0 -9.9 |  | 132 | 10.7 | 9.4 -12.2 |
| 20 - <25 | 69 | 10.2 | 8.7 -12.0 |  | 52 | 12.6 | 10.7 -14.8 |
| 25 - <30 | 180 | 10.2 | 9.2 -11.2 |  | 121 | 12.1 | 10.6 -13.8 |
| 30 - <35 | 1561 | 10.9 | 10.6 -11.3 |  | 1034 | 13.2 | 12.7 -13.7 |
| 35 - <40 | 198 | 11.3 | 10.3 -12.4 |  | 113 | 14.3 | 12.8 -16.1 |
| 40 - <45 | 273 | 10.7 | 9.9 -11.6 |  | 193 | 13.8 | 12.8 -14.9 |
| 45 - <50 | 362 | 10.9 | 10.2 -11.6 |  | 253 | 12.3 | 11.3 -13.4 |
| 50 - <60 | 92 | 10.0 | 8.8 -11.3 |  | 60 | 14.9 | 12.8 -17.4 |
| 60 - <65 | 182 | 10.6 | 9.7 -11.5 |  | 127 | 12.2 | 11.0 -13.5 |
| 65 - <80 | 105 | 10.8 | 9.6 -12.2 |  | 78 | 10.8 | 8.9 -12.9 |
| 80 - <95 | 86 | 9.3 | 8.0 -10.8 |  | 82 | 11.3 | 9.9 -12.9 |
| 95 - 120 | 80 | 8.6 | 7.5 -10.0 |  | 60 | 10.3 | 8.7 -12.2 |
| Total | 3525 |  |  |  | 2453 |  |  |
|  | | | | | | | |
| **Evening** |  | Cortisol (nmol/l) | |  |  | Cortisol (nmol/l) | |
| Clock time | n | geometric mean | 95% CI |  | n | geometric mean | 95% CI |
| 17:00 - 19:29 | 123 | 2.1 | 1.8 -2.4 |  | 96 | 2.1 | 1.8 -2.5 |
| 19:30 - 19:44 | 151 | 1.8 | 1.6 -2.1 |  | 87 | 1.8 | 1.5 -2.2 |
| 19:45 - 19:59 | 236 | 1.4 | 1.3 -1.5 |  | 167 | 1.5 | 1.3 -1.6 |
| 20:00 - 20:14 | 1588 | 1.5 | 1.4 -1.5 |  | 958 | 1.5 | 1.5 -1.6 |
| 20:15 - 20:29 | 330 | 1.4 | 1.3 -1.5 |  | 219 | 1.5 | 1.4 -1.8 |
| 20:30 - 20:44 | 292 | 1.4 | 1.3 -1.6 |  | 197 | 1.5 | 1.4 -1.7 |
| 20:45 - 20:59 | 129 | 1.3 | 1.2 -1.5 |  | 76 | 1.3 | 1.1 -1.6 |
| 21:00 - 21:29 | 301 | 1.3 | 1.2 -1.4 |  | 247 | 1.3 | 1.2 -1.4 |
| 21:30 - 21:59 | 160 | 1.2 | 1.1 -1.4 |  | 127 | 1.3 | 1.1 -1.5 |
| 22:00 - 22:14 | 157 | 1.3 | 1.1 -1.4 |  | 172 | 1.4 | 1.3 -1.6 |
| 22:15 - 22:44 | 147 | 1.5 | 1.3 -1.8 |  | 140 | 1.3 | 1.2 -1.5 |
| 22:45 - 23:14 | 129 | 1.4 | 1.2 -1.7 |  | 135 | 1.5 | 1.3 -1.7 |
| 23:15 - 23:59 | 130 | 1.7 | 1.5 -2.0 |  | 163 | 1.5 | 1.3 -1.7 |
| 24:00 - 02:30 | 8 | 1.3 | 0.9 -1.8 |  | 7 | 2.2 | 0.8 -5.9 |
| Total | 3881 |  |  |  | 2791 |  |  |

**S5 Correlations between DCS-exposure scales and between ERI-exposure scales.**

**Table S5 Spearman correlations between continuous DCS-exposure scales and between continuous ERI-exposure scales^a^**.

| **DCS** | **Control** | **Support** | **Job-strain** | **Iso-strain** |
| --- | --- | --- | --- | --- |
| **Demands** | 0.06 | 0.24 | 0.80 | 0.59 |
| **Control** |  | 0.31 | 0.59 | 0.52 |
| **Support** |  |  | 0.36 | 0.86 |
| **Job-strain** |  |  |  | 0.77 |
|  | | | | |
| **ERI** | **Reward** | **Overcom.** | **ER-ratio** | **O-ER-ratio** |
| **Effort** | 0.46 | 0.56 | 0.92 | 0.90 |
| **Reward** |  | 0.32 | 0.74 | 0.67 |
| **Overcom.** |  |  | 0.55 | 0.76 |
| **ER-ratio** |  |  |  | 0.95 |

^a^All correlations are significant at p<0.0001. N between 7130 and 7350.

Note that control, support and reward scale scores increase with decreasing levels of control, support and reward, making all correlations positive.

**S6 Four-level categorical job-strain and ER-strain exposure variables.**

**Table S6 Joint distribution of demand and control and of effort and reward by questionnaire response options and scale scores rounded to nearest integer. Cell percents (%) are percents of total.**

Colours correspond to levels of categorical job-strain and ER-strain: grey: ‘very low’, green: ’low’, orange: ‘medium’, blue: ‘high’.^a^

| **DCS-exposures** | | | **High demands** | | | | | | | | | | | |
| --- | --- | --- | --- | --- | --- | --- | --- | --- | --- | --- | --- | --- | --- | --- |
|  | Item verbal anchors and scores | | Never/almost  never | | Seldom | | Sometimes | | Often | | Always | | Total | |
|  |  |  | 1 | | 2 | | 3 | | 4 | | 5 | |  |  |
|  |  |  | n | % | n | % | n | % | n | % | n | % | N | % |
| **Low con-trol** | Never/almost never | 1 | 18 | 0.3 | 52 | 0.7 | 63 | 0.86 | 34 | 0.46 | 8 | 0.1 | 175 | 2.4 |
|  | Seldom | 2 | 122 | 1.7 | 897 | 12.2 | 1487 | 20.3 | 708 | 9.6 | 82 | 1.1 | 3296 | 44.9 |
|  | Sometimes | 3 | 123 | 1.7 | 909 | 12.4 | 1501 | 20.5 | 814 | 11.1 | 150 | 2.0 | 3497 | 47.6 |
|  | Often | 4 | 35 | 0.5 | 87 | 1.2 | 120 | 1.6 | 96 | 1.3 | 21 | 0.3 | 359 | 4.9 |
|  | Always | 5 | 3 | 0.0 | 5 | 0.1 | 5 | 0.1 | 1 | 0.0 | 0 | 0.0 | 14 | 0.2 |
|  | Total | | 301 | 4.1 | 1950 | 26.6 | 3176 | 43.3 | 1653 | 22.5 | 261 | 3.6 | 7341 | 100 |
| **ERI-exposures** | | | **High effort** | | | | | | | | | | | |
|  | Agreement with statements on high efforts and low reward | | Disagree | | Agree, I am not bothered | | Agree, I am bothered a little | | Agree, I am bothered somewhat | | Agree, I am bothered a lot | | Total | |
|  |  |  | 1 | | 2 | | 3 | | 4 | | 5 | |  |  |
|  |  |  | n | % | n | % | n | % | n | % | n | % | N | % |
| **Low re-ward** | Disagree | 1 | 432 | 6.0 | 671 | 9.3 | 321 | 4.4 | 89 | 1.2 | 7 | 0.1 | 1520 | 21.0 |
|  | Agree, I am not bothered | 2 | 403 | 5.6 | 1186 | 16.4 | 1067 | 14.7 | 504 | 7.0 | 82 | 1.1 | 3242 | 44.7 |
|  | Agree, I am bothered a little | 3 | 94 | 1.3 | 412 | 5.7 | 644 | 8.9 | 552 | 7.6 | 158 | 2.2 | 1860 | 25.6 |
|  | Agree, I am bothered somewhat | 4 | 17 | 0.2 | 74 | 1.0 | 164 | 2.3 | 205 | 2.8 | 115 | 1.6 | 575 | 7.9 |
|  | Agree, I am bothered a lot | 5 | 3 | 0.0 | 6 | 0.1 | 10 | 0.1 | 22 | 0.3 | 18 | 0.3 | 59 | 0.8 |
|  | Total | | 949 | 13.1 | 2349 | 32.4 | 2206 | 30.4 | 1372 | 18.9 | 380 | 5.2 | 7256 | 100.0 |

^a^	Job strain and ER-strain: ’very low’: one main variable has scores from 1 to <2.5, the other may take all other score values; ‘low’: both main variables have scores from 2.5 to < 3.5; ‘medium’: one main variable has scores from 2.5 to <3.5 and the other main variable has scores from 3.5 to 5; ‘high’: both main variables have scores from 3.5 to 5.

Comments:

The ‘very low’ level includes response options of no exposure or very low exposure to one of the two variables and allows any response option of the other variable. This combination is unlikely to produce an interaction which is not controlled for in analyses controlling for main effects.

Summary of composite variable distributions:

Job strain: ‘very low’ (n=4633, 63%), ‘low’ (n=1501, 20%), ‘medium’ (n=1089, 15%), ‘high (n=118, 1.6%).

ER-strain: ‘very low’ (n=5368, 74%), ‘low’ (n=644, 8.9%), ‘medium’ (n=884, 12%), ‘high (n=360, 5.0%) .

**S7.** **Influence of level of confounder control on effects of DCS- and ERI-exposures.**

Tables S7_1 to S7_6 show the influence of different levels of confounder control on effects of *continuous* DCS- and ERI-exposures. Parallel analyses of *categorical* exposures showed similar quite stable exposure effects across levels of confounder control and are therefore not shown. They can be obtained from the corresponding author.**Table S7_1. DCS continuous exposure effects on morning cortisol by increasing level of adjustment for effects of potential confounders.**

| **DCS continuous exposures and morning cortisol** | | | **Level of adjustment for potential confounders** | | | | | | | | | | | |
| --- | --- | --- | --- | --- | --- | --- | --- | --- | --- | --- | --- | --- | --- | --- |
|  |  |  | **Model 1**  **Adjustment only for**  **examination round**  **(**n=5702/5978)^c^ | | | | **Model 2**  **Adjustment for a limited set of potential confounders**^a^  (n=5326/5978)^c^ | | | | **Model 3^b^**  **Adjustment for all**  **potential confounders**  (n=5105/5978) | | | |
| **Continuous**  **exposures** | **Exposure unit**  **(range)** | **Effects**  **on**  **cortisol** | **Effect**  **Ratio**^d^ | **95% CI** | **p1^e^** | **p2^f^** | **Effect**  **Ratio**^d^ | **95% CI** | **p1^e^** | **p2^f^** | **Effect**  **Ratio**^d^ | **95% CI** | **p1^e^** | **p2^f^** |
| Demands | 1 unit (1-5) | peak | 1.06 | 0.92 -1.21 | 0.44 | 0.72 | 1.01 | 0.87 -1.17 | 0.90 | 0.91 | 1.00 | 0.87 -1.16 | 0.97 | 0.85 |
|  |  | pre-peak slope | 1.10 | 0.75 -1.60 | 0.63 |  | 1.03 | 0.68 -1.56 | 0.88 |  | 1.08 | 0.70 -1.64 | 0.73 |  |
|  |  | post-peak slope | 1.05 | 0.71 -1.56 | 0.79 |  | 1.12 | 0.73 -1.70 | 0.61 |  | 1.15 | 0.75 -1.76 | 0.52 |  |
| ntrol | 1 unit (1-5) | peak | 1.00 | 0.86 -1.17 | 0.96 | 0.65 | 0.98 | 0.83 -1.16 | 0.85 | 0.69 | 0.99 | 0.84 -1.17 | 0.88 | 0.74 |
|  |  | pre-peak slope | 0.88 | 0.56 -1.37 | 0.56 |  | 0.85 | 0.52 -1.37 | 0.50 |  | 0.96 | 0.58 -1.59 | 0.86 |  |
|  |  | post-peak slope | 1.21 | 0.77 -1.88 | 0.41 |  | 1.25 | 0.78 -1.99 | 0.36 |  | 1.27 | 0.79 -2.03 | 0.33 |  |
| Support | 1 unit (1-5) | peak | 1.02 | 0.94 -1.09 | 0.68 | 0.96 | 1.01 | 0.93 -1.08 | 0.88 | 0.98 | 1.02 | 0.95 -1.10 | 0.54 | 0.93 |
|  |  | pre-peak slope | 0.99 | 0.80 -1.24 | 0.96 |  | 0.98 | 0.78 -1.23 | 0.83 |  | 1.03 | 0.82 -1.30 | 0.79 |  |
|  |  | post-peak slope | 0.96 | 0.78 -1.18 | 0.71 |  | 1.00 | 0.80 -1.24 | 0.99 |  | 0.99 | 0.79 -1.23 | 0.91 |  |
| Job-strain | 1 unit (0.20-5) | peak | 0.92 | 0.67 -1.26 | 0.60 | 0.83 | 0.96 | 0.68 -1.34 | 0.79 | 0.91 | 0.99 | 0.70 -1.39 | 0.94 | 0.92 |
|  |  | pre-peak slope | 0.87 | 0.35 -2.17 | 0.76 |  | 0.86 | 0.32 -2.32 | 0.77 |  | 0.84 | 0.30 -2.35 | 0.74 |  |
|  |  | post-peak slope | 0.86 | 0.35 -2.12 | 0.74 |  | 0.82 | 0.32 -2.14 | 0.69 |  | 0.81 | 0.31 -2.11 | 0.66 |  |
| Iso-strain | 1 unit (0.04-5) | peak | 1.02 | 0.81 -1.28 | 0.85 | 0.95 | 1.06 | 0.84 -1.35 | 0.61 | 0.89 | 1.03 | 0.81 -1.30 | 0.82 | 1.00 |
|  |  | pre-peak slope | 1.18 | 0.61 -2.30 | 0.62 |  | 1.31 | 0.66 -2.63 | 0.44 |  | 1.08 | 0.54 -2.17 | 0.83 |  |
|  |  | post-peak slope | 1.05 | 0.55 -2.01 | 0.89 |  | 0.94 | 0.48 -1.84 | 0.85 |  | 0.94 | 0.47 -1.85 | 0.85 |  |

^a^ Model 2: examination round, age, gender, pregnancy, education, personal income, saliva samples taken on a workday vs. on a day-off, and fixed daytime work schedule.

^b^ Model 3: analytic model as described in Supplementary material S2, potential confounders as described in the main text section 2.4, plus pre- and post-peak times for morning cortisol, and all pre- and post- peak and nadir times for evening cortisol and morning-to-evening slopes.

^c^ Number of observations used in the analyses out of all observations with valid information on cortisol concentrations and relevant saliva sampling time information

^d^ Effect ratios are the ratios by which the peak or nadir cortisol concentrations and corresponding slopes change with a one unit change of exposure or for an exposure category compared to the reference category. The unit of peak or nadir concentrations is nmol/l saliva. The unit of slopes is nmol/l saliva per time unit. Slope time units are all one hour except that the pre-peak slope unit is set to half an hour. Effect ratios above unity implies that peak and nadir cortisol concentrations increase, increasing slopes (pre-peak and post-nadir slopes) become steeper, and decreasing slopes (post-peak slopes and pre-nadir slopes) become flatter (and vice versa).

^e^ p1: probability that the estimated effect ratio differs from unity (test with one degree of freedom).

^f^ p2: probability that estimated exposure effects on peak or nadir concentrations and associated slopes, considered together, differs from no effect. Degrees of freedom is the number of estimated effect ratios.

**Table S7_2. DCS continuous exposure effects on evening cortisol by increasing level of adjustment for effects of potential confounders.**

| **DCS continuous exposures and evening cortisol** | | | **Level of adjustment for potential confounders** | | | | | | | | | | | |
| --- | --- | --- | --- | --- | --- | --- | --- | --- | --- | --- | --- | --- | --- | --- |
|  |  |  | **Model 1**  **Adjustment only for**  **examination round**  **(**n=5936/6230)^c^ | | | | **Model 2**  **Adjustment for a limited set of potential.**  **confounders**^a^  (n=5541/6230)^c^ | | | | **Model 3^b^**  **Adjustment for all**  **potential confounders**  (n=5317/6230)^c^ | | | |
| **Continuous**  **exposures** | **Exposure unit**  **(range)** | **Effects**  **on**  **cortisol** | **Effect**  **Ratio**^d^ | **95% CI** | **p1^e^** | **p2^f^** | **Effect**  **Ratio**^d^ | **95% CI** | **p1^e^** | **p2^f^** | **Effect**  **Ratio**^d^ | **95% CI** | **p1^e^** | **p2^f^** |
| Demands | 1 unit (1-5) | nadir | 0.88 | 0.70 -1.10 | 0.26 | 0.61 | 0.85 | 0.67 -1.08 | 0.19 | 0.55 | 0.90 | 0.71 -1.15 | 0.41 | 0.80 |
|  |  | pre-nadir slope | 0.88 | 0.69 -1.14 | 0.33 |  | 0.86 | 0.66 -1.12 | 0.27 |  | 0.88 | 0.67 -1.15 | 0.34 |  |
|  |  | post-nadir slope | 1.02 | 0.85 -1.22 | 0.86 |  | 1.04 | 0.86 -1.26 | 0.70 |  | 1.03 | 0.85 -1.26 | 0.76 |  |
| Control | 1 unit (1-5) | nadir | 0.88 | 0.68 -1.13 | 0.32 | 0.20 | 0.86 | 0.66 -1.12 | 0.26 | 0.25 | 0.89 | 0.68 -1.17 | 0.41 | 0.45 |
|  |  | pre-nadir slope | 0.91 | 0.69 -1.20 | 0.50 |  | 0.89 | 0.67 -1.19 | 0.44 |  | 0.88 | 0.65 -1.19 | 0.40 |  |
|  |  | post-nadir slope | 0.92 | 0.75 -1.20 | 0.43 |  | 0.94 | 0.76 -1.17 | 0.59 |  | 0.94 | 0.75 -1.18 | 0.60 |  |
| Support | 1 unit (1-5) | nadir | 1.01 | 0.90 -1.14 | 0.82 | 0.97 | 1.01 | 0.89 -1.15 | 0.84 | 0.82 | 1.00 | 0.88 -1.13 | 0.97 | 0.74 |
|  |  | pre-nadir slope | 0.99 | 0.87 -1.12 | 0.83 |  | 0.96 | 0.84 -1.10 | 0.56 |  | 0.95 | 0.84 -1.09 | 0.50 |  |
|  |  | post-nadir slope | 1.01 | 0.91 -1.12 | 0.84 |  | 1.01 | 0.91 -1.12 | 0.92 |  | 1.00 | 0.90 -1.11 | 0.97 |  |
| Job-strain | 1 unit (0.20-5) | nadir | 1.24 | 0.73 -2.11 | 0.42 | 0.54 | 1.32 | 0.76 -2.31 | 0.33 | 0.52 | 1.18 | 0.66 -2.09 | 0.58 | 0.84 |
|  |  | pre-nadir slope | 1.17 | 0.65 -2.10 | 0.60 |  | 1.18 | 0.64 -2.21 | 0.59 |  | 1.15 | 0.61 -2.17 | 0.67 |  |
|  |  | post-nadir slope | 1.09 | 0.71 -1.68 | 0.68 |  | 1.04 | 0.66 -1.62 | 0.87 |  | 1.05 | 0.66 -1.66 | 0.85 |  |
| Iso-strain | 1 unit (0.04-5) | nadir | 0.97 | 0.66 1.42- | 0.88 | 0.77 | 0.97 | 0.65 -1.43 | 0.87 | 0.53 | 1.03 | 0.69 -1.54 | 0.87 | 0.68 |
|  |  | pre-nadir slope | 1.07 | 0.71 -1.61 | 0.76 |  | 1.15 | 0.75 -1.77 | 0.52 |  | 1.20 | 0.78 -1.84 | 0.42 |  |
|  |  | post-nadir slope | 0.93 | 0.68 -1.27 | 0.65 |  | 0.94 | 0.68 -1.30 | 0.72 |  | 0.94 | 0.68 -1.31 | 0.72 |  |

^a - f^ See footnotes to Table S7_1

**Table S7_3. ERI continuous exposure effects on morning cortisol by increasing level of adjustment for effects of potential confounders.**

| **ERI continuous exposures and morning cortisol** | | | **Level of adjustment for potential confounders** | | | | | | | | | | | |
| --- | --- | --- | --- | --- | --- | --- | --- | --- | --- | --- | --- | --- | --- | --- |
|  |  |  | **Model 1**  **Adjustment only for**  **examination round**  **(**n=5676/5978)^c^ | | | | **Model 2**  **Adjustment for a limited set of potential.**  **confounders**^a^  (n=5299/5978)^c^ | | | | **Model 3^b^**  **Adjustment for all**  **potential confounders**  (n=5078/5978)^c^ | | | |
| **Continuous**  **exposures** | **Exposure unit**  **(range)** | **Effects**  **on**  **cortisol** | **Effect**  **Ratio**^d^ | **95% CI** | **p1^e^** | **p2^f^** | **Effect**  **Ratio**^d^ | **95% CI** | **p1^e^** | **p2^f^** | **Effect**  **Ratio**^d^ | **95% CI** | **p1^e^** | **p2^f^** |
| Effort | 1 unit (1-5) | peak | 1.02 | 0.94 -1.11 | 0.62 | 0.97 | 1.01 | 0.93 -1.11 | 0.76 | 0.90 | 1.03 | 0.95 -1.13 | 0.48 | 0.66 |
|  |  | pre-peak slope | 1.05 | 0.84 -1.31 | 0.68 |  | 0.97 | 0.77 -1.23 | 0.82 |  | 0.98 | 0.77 -1.24 | 0.86 |  |
|  |  | post-peak slope | 0.97 | 0.78 -1.22 | 0.82 |  | 1.04 | 0.82 -1.31 | 0.77 |  | 1.04 | 0.82 -1.31 | 0.74 |  |
| Reward | 1 unit (1-5) | peak | 0.98 | 0.82 -1.17 | 0.79 | 0.96 | 0.96 | 0.79 -1.15 | 0.63 | 0.73 | 1.02 | 0.84 -1.23 | 0.86 | 0.50 |
|  |  | pre-peak slope | 0.96 | 0.55 -1.66 | 0.88 |  | 0.83 | 0.46 -1.47 | 0.52 |  | 0.80 | 0.45 -1.43 | 0.46 |  |
|  |  | post-peak slope | 1.16 | 0.69 -1.94 | 0.58 |  | 1.33 | 0.78 -2.26 | 0.29 |  | 1.28 | 0.75 2.18- | 0.36 |  |
| Overcom. | 1 unit (1-5) | peak | 1.00 | 0.92 -1.09 | 0.98 | 0.10 | 0.99 | 0.90 -1.08 | 0.81 | 0.09 | 1.00 | 0.91 -1.09 | 0.93 | 0.45 |
|  |  | pre-peak slope | 1.01 | 0.80 -1.27 | 0.96 |  | 0.97 | 0.76 -1.23 | 0.77 |  | 0.95 | 0.75 -1.22 | 0.70 |  |
|  |  | post-peak slope | 0.76 | 0.60 -0.97 | **0.03** |  | 0.77 | 0.60 -0.99 | **0.04** |  | 0.84 | 0.65 -1.09 | 0.19 |  |
| ER-ratio | 1 unit (0.20-5) | peak | 1.03 | 0.68 -1.56 | 0.88 | 0.70 | 1.05 | 0.69 -1.60 | 0.82 | 0.49 | 0.91 | 0.60 -1.40 | 0.68 | 0.52 |
|  |  | pre-peak slope | 1.08 | 0.41 -2.84 | 0.88 |  | 1.33 | 0.49 -3.64 | 0.58 |  | 1.12 | 0.41 -3.07 | 0.82 |  |
|  |  | post-peak slope | 0.58 | 0.22 -1.54 | 0.27 |  | 0.50 | 0.18 -1.35 | 0.17 |  | 0.62 | 0.23 -1.68 | 0.35 |  |
| O-ER-ratio | 1 unit (0.04-5) | peak | 0.93 | 0.66 -1.30 | 0.66 | 0.19 | 0.95 | 0.67 -1.35 | 0.77 | 0.26 | 1.06 | 0.75 -1.51 | 0.74 | 0.55 |
|  |  | pre-peak slope | 0.84 | 0.39 -1.81 | 0.66 |  | 0.86 | 0.39 -1.91 | 0.71 |  | 1.18 | 0.52 -2.70 | 0.69 |  |
|  |  | post-peak slope | 2.36 | 1.04 -5.38 | **0.04** |  | 2.20 | 0.94 -5.11 | 0.07 |  | 1.55 | 0.65 -3.68 | 0.32 |  |

**^a – f^** See footnotes to Table S7_1

**Table S7_4. ERI continuous exposure effects on evening cortisol by increasing level of adjustment for effects of potential confounders.**

| **ERI continuous exposures and evening cortisol** | | | **Level of adjustment for potential confounders** | | | | | | | | | | | |
| --- | --- | --- | --- | --- | --- | --- | --- | --- | --- | --- | --- | --- | --- | --- |
|  |  |  | **Model 1**  **Adjustment only for**  **examination round**  **(**n=5903/6230)^c^ | | | | **Model 2**  **Adjustment for a limited set of potential.**  **confounders**^a^  (n=5496/6230)^c^ | | | | **Model 3^b^**  **Adjustment for all**  **potential confounders**  (n=5285/6230)^c^ | | | |
| **Continuous**  **exposures** | **Exposure unit**  **(range)** | **Effects**  **on**  **cortisol** | **Effect**  **Ratio**^d^ | **95% CI** | **p1^e^** | **p2^f^** | **Effect**  **Ratio**^d^ | **95% CI** | **p1^e^** | **p2^f^** | **Effect**  **Ratio**^d^ | **95% CI** | **p1^e^** | **p2^f^** |
| Effort | 1 unit (1-5) | nadir | 0.98 | 0.86 -1.12 | 0.79 | 0.72 | 0.98 | 0.85 -1.12 | 0.74 | 0.51 | 1.00 | 0.86 -1.17 | 0.96 | 0.52 |
|  |  | pre-nadir slope | 0.97 | 0.85 -1.11 | 0.69 |  | 0.95 | 0.83 -1.09 | 0.50 |  | 0.98 | 0.82 -1.16 | 0.78 |  |
|  |  | post-nadir slope | 0.96 | 0.85 -1.08 | 0.49 |  | 0.95 | 0.84 -1.07 | 0.40 |  | 0.93 | 0.82 -1.06 | 0.28 |  |
| Reward | 1 unit (1-5) | nadir | 0.97 | 0.75 -1.26 | 0.85 | 0.88 | 0.99 | 0.76 -1.29 | 0.94 | 0.86 | 0.99 | 0.71 -1.37 | 0.94 | 0.86 |
|  |  | pre-nadir slope | 1.04 | 0.82 -1.30 | 0.77 |  | 1.02 | 0.81 -1.29 | 0.87 |  | 1.01 | 0.69 -1.47 | 0.97 |  |
|  |  | post-nadir slope | 0.96 | 0.75 -1.24 | 0.78 |  | 0.92 | 0.71 -1.20 | 0.53 |  | 0.92 | 0.69 -1.22 | 0.54 |  |
| Overcom. | 1 unit (1-5) | nadir | 0.94 | 0.80 -1.09 | 0.41 | 0.77 | 0.92 | 0.78 -1.08 | 0.29 | 0.63 | 0.96 | 0.81 -1.13 | 0.61 | 0.62 |
|  |  | pre-nadir slope | 0.95 | 0.80 -1.12 | 0.51 |  | 0.95 | 0.80 -1.12 | 0.53 |  | 1.01 | 0.84 -1.20 | 0.93 |  |
|  |  | post-nadir slope | 1.07 | 0.94 -1.21 | 0.29 |  | 1.08 | 0.95 -1.23 | 0.25 |  | 1.06 | 0.93 -1.21 | 0.36 |  |
| ER-ratio | 1 unit (0.20-5) | nadir | 0.93 | 0.49 -1.77 | 0.83 | 0.96 | 0.94 | 0.49 -1.82 | 0.86 | 0.87 | 0.99 | 0.48 -2.04 | 0.97 | 0.84 |
|  |  | pre-nadir slope | 0.99 | 0.52 -1.90 | 0.98 |  | 1.06 | 0.54 -2.07 | 0.87 |  | 1.15 | 0.50 -2.64 | 0.75 |  |
|  |  | post-nadir slope | 1.16 | 0.64 -2.08 | 0.62 |  | 1.21 | 0.66 -2.23 | 0.53 |  | 1.19 | 0.64 -2.24 | 0.58 |  |
| O-ER-ratio | 1 unit (0.04-5) | nadir | 1.12 | 0.63 -2.01 | 0.70 | 0.93 | 1.12 | 0.62 -2.05 | 0.70 | 0.91 | 0.96 | 0.52 -1.78 | 0.90 | 0.76 |
|  |  | pre-nadir slope | 1.03 | 0.54 -1.95 | 0.93 |  | 0.99 | 0.51 -1.94 | 0.98 |  | 0.81 | 0.40 -1.62 | 0.55 |  |
|  |  | post-nadir slope | 0.92 | 0.57 -1.47 | 0.72 |  | 0.92 | 0.56 -1.49 | 0.73 |  | 0.99 | 0.61 -1.62 | 0.98 |  |

**^a – f^** See footnotes to Table S7_1

**Table S7_5. DCS continuous exposure effects on morning-to-evening cortisol slope by increasing level of adjustment for effects of potential confounders.**

| **DCS**  **Morning-to-evening**  **cortisol slope** | | **Level of adjustment for potential confounders** | | | | | | | | | | | |
| --- | --- | --- | --- | --- | --- | --- | --- | --- | --- | --- | --- | --- | --- |
|  |  | **Model 1**  **Adjustment only for**  **examination round**  (n=5542/5811)^c^ | | | | **Model 2^a^**  **Adjustment for a limited set of potential.**  **confounders**^a^  (n=5178/5811)^c^ | | | | **Model 3^b^**  **Adjustment for all**  **potential confounders**  (n=4969/5811)^c^ | | | |
| **Continuous exposures** | | | | | | | | | | | | | |
|  | **Exposure unit**  **(score range)** | **Effect**  **ratio**^d^ | **95% CI** | **p1^e^** |  | **Effect**  **ratio**^d^ | **95% CI** | **p1^e^** |  | **Effect**  **ratio**^d^ | **95% CI** | **p1^e^** |  |
| Demands | 1 unit (1-5) | 1.12 | 0.96 -1.30 | 0.15 |  | 1.10 | 0.93 -1.28 | 0.26 |  | 1.04 | 0.88 - 1.22 | 0.66 |  |
| Control | 1 unit (1-5) | 1.19 | 1.00 -1.40 | 0.05 |  | 1.18 | 0.99 -1.41 | 0.07 |  | 1.11 | 0.92-1.33 | 0.27 |  |
| Support | 1 unit (1-5) | 0.99 | 0.91 -1.07 | 0.79 |  | 0.98 | 0.90 -1.06 | 0.56 |  | 1.00 | 0.92 - 1.09 | 0.96 |  |
| Job-strain | 1 unit (0.20-5) | 0.77 | 0.54 -1.09 | 0.14 |  | 0.77 | 0.53 -1.11 | 0.16 |  | 0.87 | 0.60-1.28 | 0.49 |  |
| Iso-strain | 1 unit (0.04-5) | 1.09 | 0.85 -1.40 | 0.50 |  | 1.15 | 0.89 -1.50 | 0.29 |  | 1.09 | 0.83 -1.42 | 0.53 |  |

^a, b, c ,e^ See footnotes to Tables S7_1 to S7_4 at the start of this section (S7).

^d^ Effect ratios are the ratios by which the morning-to-evening geometric mean cortisol concentration ratio change with a one unit change of exposure. Effect ratios above unity implies that the morning-to-evening slope becomes steeper.

**Table S7_6. ERI continuous exposure effects on morning-to-evening cortisol slope by increasing level of adjustment for effects of potential confounders.**

| **ERI**  **Morning-to-evening**  **cortisol slope** | | **Level of adjustment for potential confounders** | | | | | | | | | | | |
| --- | --- | --- | --- | --- | --- | --- | --- | --- | --- | --- | --- | --- | --- |
|  |  | **Model 1**  **Adjustment only for**  **examination round**  (n=5515/5811)^c^ | | | | **Model 2^a^**  **Adjustment for a limited set of potential.**  **confounders**^a^  (n=5150/5811)^c^ | | | | **Model 3^b^**  **Adjustment for all**  **potential confounders**  (n= 4941/5811)^c^ | | | |
| **Continuous exposures** | | | | | | | | | | | | | |
|  | **Exposure unit**  **(score range)** | **Effect**  **ratio**^d^ | **95% CI** | **p1^e^** |  | **Effect**  **ratio**^d^ | **95% CI** | **p1^e^** |  | **Effect**  **ratio**^d^ | **95% CI** | **p1^e^** |  |
| Effort | 1 unit (1-5) | 1.02 | 0.93 -1.12 | 0.66 |  | 1.03 | 0.93 -1.13 | 0.60 |  | 1.04 | 0.94 -1.15 | 0.43 |  |
| Rewards | 1 unit (1-5) | 1.06 | 0.86 -1.30 | 0.61 |  | 1.06 | 0.86 -1.32 | 0.57 |  | 1.13 | 0.91 -1.40 | 0.27 |  |
| Overcom. | 1 unit (1-5) | 0.98 | 0.89 -1.08 | 0.69 |  | 0.99 | 0.89 -1.10 | 0.85 |  | 1.02 | 0.92 -1.13 | 0.74 |  |
| ER-ratio | 1 unit (0.20-5) | 1.03 | 0.65 -1.64 | 0.91 |  | 1.01 | 0.63 -1.63 | 0.97 |  | 0.94 | 0.58 -1.53 | 0.81 |  |
| O-ER-ratio | 1 unit (0.04-5) | 0.93 | 0.63 -1.64 | 0.69 |  | 0.92 | 0.62 -1.36 | 0.68 |  | 0.92 | 0.62 -1.36 | 0.67 |  |

^-f^ See footnotes to Table S7_5

**S8. Sensitivity analyses of possible effect attenuation due to collinearity**

Composite exposures were highly correlated with one or more of their constituent main exposures (Supplementary material S5). Consequently, some exposure effect estimates could be attenuated, and meaningful significant results could be missed. We therefore made a set of analyses corresponding to the main analyses of morning and evening cortisol and of the peak-to-nadir cortisol slope where highly correlated exposures were not included in the same statistical model:

**Model 1**: included the three main exposures, composite variables excluded.

**Model 2:** included the two-way composite variable as defined in the main text. Main exposures and the three-way composite variable were excluded.

**Model 3**: included the three-way composite variable as defined in the text. Main exposures and the two-way composite variable were excluded).

Effects of exposure variables in Model 1 were mutually adjusted. All exposure effects were adjusted for relevant saliva sampling times (see Table footnotes) and all other potential confounders.

In summary, these analyses showed a few sporadic low-level significant effect ratios of specific exposure-outcome relations (0.01<p<0.05) but there were no overall significant effects of main exposures or of the two-way or the three-way composite variables. Thus, these analyses did not indicate that the main analyses missed any important information due to attenuation caused by collinearity between composite exposures and their constituent main exposures or between the two- and three-way composite exposures.

**Table S8_1a. Effects of DCS continuous exposures on morning and evening cortisol patterns. By exposure models 1 to 3.**

| **DCS continuous exposures** | | **Morning cortisol^a^**  (n= 5105/5978)^c^ | | | |  | **Evening cortisol**^b^  (n=5317/6230)^c^ | | | |
| --- | --- | --- | --- | --- | --- | --- | --- | --- | --- | --- |
| **Exposure** | **Effect on cortisol** | **Effect**  **Ratio^d^** | **95% CI** | **p1^e^** | **p2^f^** | **Effect on cortisol** | **Effect**  **Ratio^d^** | **95% CI** | **p1^e^** | **p2^f^** |
| **Model 1** | | | | | | | | | | |
| Demands | peak | 1.00 | 0.97 -1.04 | 0.85 | 0.99 | nadir | 0.99 | 0.94 -1.04 | 0.62 | 0.22 |
|  | pre-peak slope | 1.01 | 0.92 -1.11 | 0.89 |  | pre-nadir slope | 0.98 | 0.92 -1.03 | 0.42 |  |
|  | post-peak slope | 1.01 | 0.92 -1.10 | 0.86 |  | post-nadir slope | 1.04 | 1.00 -1.09 | 0.07 |  |
| Control | peak | 0.99 | 0.94 -1.04 | 0.61 | 0.20 | nadir | 0.98 | 0.91 -1.07 | 0.71 | 0.17 |
|  | pre-peak slope | 0.88 | 0.76 -1.02 | 0.10 |  | pre-nadir slope | 0.99 | 0.91 -1.08 | 0.87 |  |
|  | post-peak slope | 1.10 | 0.96 -1.26 | 0.15 |  | post-nadir slope | 0.95 | 0.89 -1.02 | 0.14 |  |
| Support | peak | 1.03 | 1.00 -1.06 | 0.03 | 0.18 | nadir | 1.01 | 0.96 -1.06 | 0.81 | 0.86 |
|  | pre-peak slope | 1.06 | 0.97 -1.15 | 0.21 |  | pre-nadir slope | 1.00 | 0.95 -1.06 | 0.90 |  |
|  | post-peak slope | 0.97 | 0.90 -1.04 | 0.40 |  | post-nadir slope | 0.98 | 0.95 -1.02 | 0.44 |  |
| **Model 2** | | | | | | | | | | |
| Job-strain | peak | 1.02 | 0.97 -1.06 | 0.48 | 0.34 | nadir | 0.99 | 0.91 -1.07 | 0.76 | 0.98 |
|  | pre-peak slope | 0.96 | 0.84 -1.10 | 0.56 |  | pre-nadir slope | 0.99 | 0.90 -1.07 | 0.74 |  |
|  | post-peak slope | 1.04 | 0.92-1.18 | 0.49 |  | post-nadir slope | 1.00 | 0.94 -1.06 | 0.98 |  |
| **Model 3** | | | | | | | | | | |
| Iso-strain | peak | 1.05 | 0.99 -1.11 | 0.08 | 0.17 | nadir | 1.00 | 0.91 -1.09 | 0.97 | 0.82 |
|  | pre-peak slope | 1.02 | 0.87 -1.19 | 0.83 |  | pre-nadir slope | 1.00 | 0.91 -1.10 | 0.98 |  |
|  | post-peak slope | 1.00 | 0.87 -1.15 | 1.00 |  | post-nadir slope | 0.97 | 0.91 -1.05 | 0.47 |  |

^a^ Exposure effects adjusted for early- and late waking times, pre- and post-peak time-splines, and all other potential confounders (excluding pre- and post-nadir time-splines).

^b^ Exposure effects adjusted for early- and late waking times, pre- and post-peak time-splines, pre- and post-nadir time-splines, and all other potential confounders.

^c^ Number of observations used in the analyses out of all observations with valid information on cortisol concentrations and relevant saliva sampling time information

^d^ Effect ratios are the ratios by which the peak and nadir and associated slopes change with a one unit change of exposure.

The unit of peak and nadir is nmol/l saliva. The unit of slopes is nmol/l saliva per time unit. Slope time units are all one hour except that the pre-peak slope unit is set to half an hour.
Effect ratios above unity implies that peak and nadir concentrations increase, increasing slopes (pre-peak and post-nadir slopes) become steeper, and decreasing slopes (post-peak and pre-nadir slopes) become flatter (and vice versa).

^e^ p1: probability that the observed effect ratio differs from unity (test with one degree of freedom).

^f^ p2: probability that estimated exposure effects on peak or nadir and associated slopes, considered together, differs from no effect. Degrees of freedom is the number of estimated effect ratios.

**Table S8_1b. Effects of DCS categorical exposures on morning and evening cortisol patterns. By exposure models 1 to 3.**

| **DCS categorical**  **exposures** | | **Morning cortisol**^a^  (n= 5105/5978)^c^ | | | | | **Evening cortisol^b^**  (n=5317/6230)^c^ | | | | |
| --- | --- | --- | --- | --- | --- | --- | --- | --- | --- | --- | --- |
| **Exposure** | **Exposure category** | **Exposure effect**  **on**  **cortisol** | **Effect**  **Ratio**^d^ | **95% CI** | **p1**^e^ | **p2**^f^ | **Exposure effect**  **on**  **cortisol** | **Effect**  **Ratio**^d^ | **95% CI** | **p1**^e^ | **p2**^f^ |
| **Model 1** | | | | | | | | | | | |
| Demands | medium  vs  low | peak | 1.01 | 0.96 -1.07 | 0.64 | 0.89 | nadir | 1.00 | 0.91 -1.11 | 0.96 | 0.14 |
|  |  | pre-peak slope | 1.06 | 0.88 -1.28 | 0.51 |  | pre-nadir slope | 0.96 | 0.86 -1.07 | 0.46 |  |
|  |  | post-peak slope | 0.93 | 0.79 -1.10 | 0.39 |  | post-nadir slope | 1.01 | 0.93 -1.10 | 0.78 |  |
|  | high  vs  low | peak | 1.02 | 0.95 -1.09 | 0.61 |  | nadir | 0.94 | 0.84 -1.06 | 0.32 |  |
|  |  | pre-peak slope | 1.05 | 0.85 -1.30 | 0.64 |  | pre-nadir slope | 0.92 | 0.81 -1.04 | 0.17 |  |
|  |  | post-peak slope | 1.02 | 0.84 -1.23 | 0.86 |  | post-nadir slope | 1.12 | 1.02 -1.23 | 0.02 |  |
| Control | medium  vs  high | peak | 1.02 | 0.97 -1.07 | 0.55 | 0.28 | nadir | 0.97 | 0.88 -1.05 | 0.43 | 0.50 |
|  |  | pre-peak slope | 0.90 | 0.76 -1.06 | 0.19 |  | pre-nadir slope | 0.96 | 0.87 -1.05 | 0.34 |  |
|  |  | post-peak slope | 0.99 | 0.85 -1.14 | 0.84 |  | post-nadir slope | 0.98 | 0.91 -1.06 | 0.65 |  |
|  | low  vs  high | peak | 0.92 | 0.81 -1.05 | 0.21 |  | nadir | 1.06 | 0.86 -1.31 | 0.57 |  |
|  |  | pre-peak slope | 0.69 | 0.47 -1.00 | 0.05 |  | pre-nadir slope | 1.15 | 0.90 -1.47 | 0.27 |  |
|  |  | post-peak slope | 1.14 | 0.82 -1.58 | 0.42 |  | post-nadir slope | 0.91 | 0.77 -1.06 | 0.23 |  |
| Support | medium  vs  high | peak | 1.04 | 0.96 -1.12 | 0.33 | 0.78 | nadir | 1.12 | 0.99 -1.27 | 0.08 | 0.11 |
|  |  | pre-peak slope | 1.08 | 0.85 -1.36 | 0.53 |  | pre-nadir slope | 1.07 | 0.93 -1.23 | 0.36 |  |
|  |  | post-peak slope | 0.90 | 0.74 -1.09 | 0.28 |  | post-nadir slope | 0.91 | 0.82 -1.01 | 0.08 |  |
|  | low  vs  high | peak | 1.12 | 0.96 -1.32 | 0.16 |  | nadir | 0.83 | 0.63 -1.08 | 0.17 |  |
|  |  | pre-peak slope | 1.32 | 0.82 -2.14 | 0.26 |  | pre-nadir slope | 0.96 | 0.72 -1.28 | 0.79 |  |
|  |  | post-peak slope | 0.83 | 0.50 -1.36 | 0.45 |  | post-nadir slope | 1.02 | 0.82 -1.26 | 0.87 |  |

(continues on next page)

**Table S8_1b (continued). Effects of DCS categorical exposures on morning and evening cortisol patterns. By exposure models 1 to 3.**

| **DCS categorical**  **exposures** | | **Morning cortisol**^a^  (n= 5105/5978)^c^ | | | | | **Evening cortisol^b^**  (n=5317/6230)^c^ | | | | |
| --- | --- | --- | --- | --- | --- | --- | --- | --- | --- | --- | --- |
| **Exposure** | **Exposure category** | **Exposure effect**  **on**  **cortisol** | **Effect**  **Ratio**^d^ | **95% CI** | **p1**^e^ | **p2**^f^ | **Exposure effect**  **on**  **cortisol** | **Effect**  **Ratio**^d^ | **95% CI** | **p1**^e^ | **p2**^f^ |
| **Model 2** | | | | | | | | | | | |
| Job-strain | low  vs  very low | peak | 1.00 | 0.94 -1.06 | 0.97 | 0.81 | nadir | 0.98 | 0.88 -1.09 | 0.71 | 0.77 |
|  |  | pre-peak slope | 0.91 | 0.76 -1.11 | 0.36 |  | pre-nadir slope | 0.94 | 0.84 -1.05 | 0.28 |  |
|  |  | post-peak slope | 0.97 | 0.82 -1.15 | 0.73 |  | post-nadir slope | 1.02 | 0.94 -1.12 | 0.60 |  |
|  | medium  vs  very low | peak | 1.04 | 0.97 -1.12 | 0.23 |  | nadir | 1.03 | 0.91 -1.15 | 0.66 |  |
|  |  | pre-peak slope | 0.95 | 0.77 -1.17 | 0.61 |  | pre-nadir slope | 1.07 | 0.94 -1.21 | 0.31 |  |
|  |  | post-peak slope | 0.97 | 0.81 -1.17 | 0.78 |  | post-nadir slope | 0.98 | 0.89 -1.07 | 0.61 |  |
|  | high  vs  very low | peak | 0.93 | 0.75 -1.15 | 0.50 |  | nadir | 0.91 | 0.61 -1.37 | 0.66 |  |
|  |  | pre-peak slope | 0.81 | 0.46 -1.41 | 0.46 |  | pre-nadir slope | 1.01 | 0.55 -1.83 | 0.98 |  |
|  |  | post-peak slope | 1.11 | 0.64 -1.91 | 0.71 |  | post-nadir slope | 1.07 | 0.84 -1.37 | 0.59 |  |
| **Model 3** | | | | | | | | | | | |
| Iso-strain | low  vs  very low | peak | 1.04 | 0.92 -1.18 | 0.49 | 0.44 | nadir | 0.93 | 0.74 -1.16 | 0.50 | 0.88 |
|  |  | pre-peak slope | 1.03 | 0.72 -1.46 | 0.88 |  | pre-nadir slope | 0.94 | 0.73 -1.21 | 0.62 |  |
|  |  | post-peak slope | 0.73 | 0.53 -1.02 | 0.06 |  | post-nadir slope | 1.01 | 0.84 -1.22 | 0.93 |  |
|  | medium  vs  very low | peak | 1.06 | 0.95 -1.19 | 0.28 |  | nadir | 1.13 | 0.94 -1.35 | 0.19 |  |
|  |  | pre-peak slope | 0.93 | 0.67 -1.31 | 0.69 |  | pre-nadir slope | 1.19 | 0.98 -1.45 | 0.08 |  |
|  |  | post-peak slope | 0.99 | 0.72 -1.36 | 0.95 |  | post-nadir slope | 0.92 | 0.80 -1.05 | 0.22 |  |
|  | high  vs  very low | peak | 0.93 | 0.69 -1.26 | 0.63 |  | nadir | 1.03 | 0.59 -1.81 | 0.91 |  |
|  |  | pre-peak slope | 0.53 | 0.21 -1.35 | 0.18 |  | pre-nadir slope | 1.18 | 0.53 -2.64 | 0.69 |  |
|  |  | post-peak slope | 1.39 | 0.64 -3.01 | 0.40 |  | post-nadir slope | 1.02 | 0.69 -1.51 | 0.92 |  |

^a-f^ See footnotes to Table S8_1a

**Table S8_2a Effects of ERI continuous exposures on morning and evening cortisol patterns. By exposure models 1 to 3.**

| **ERI continuous exposures** | | **Morning cortisol**^a^  (n=5078/5978)^c^ | | | |  | **Evening cortisol**^b^  (n=5285/6230)^c^ | | | |
| --- | --- | --- | --- | --- | --- | --- | --- | --- | --- | --- |
| **Exposure** | **Effect on cortisol** | **Effect**  **Ratio**^d^ | **95% CI** | **p1^e^** | **p2**^f^ | **Effect on cortisol** | **Effect**  **Ratio**^d^ | **95% CI** | **p1^e^** | **p2**^f^ |
| **Model 1** | | | | | | | | | | |
| Effort | peak | 1.02 | 0.99 -1.05 | 0.25 | 0.66 | nadir | 1.00 | 0.94 -1.05 | 0.91 | 0.32 |
|  | pre-peak slope | 1.05 | 0.96 -1.16 | 0.29 |  | pre-nadir slope | 0.98 | 0.93 -1.04 | 0.52 |  |
|  | post-peak slope | 0.97 | 0.89 -1.06 | 0.50 |  | post-nadir slope | 0.98 | 0.93 -1.02 | 0.27 |  |
| Reward | peak | 0.99 | 0.95 -1.02 | 0.45 | 0.39 | nadir | 0.97 | 0.92 -1.03 | 0.32 | 0.48 |
|  | pre-peak slope | 0.93 | 0.84 -1.03 | 0.14 |  | pre-nadir slope | 1.00 | 0.94 -1.06 | 0.88 |  |
|  | post-peak slope | 1.06 | 0.97 -1.16 | 0.23 |  | post-nadir slope | 1.02 | 0.98 -1.07 | 0.32 |  |
| Overcommitment | peak | 1.01 | 0.95 -1.06 | 0.81 | 0.87 | nadir | 0.95 | 0.87 -1.04 | 0.31 | 0.51 |
|  | pre-peak slope | 0.98 | 0.84 -1.15 | 0.84 |  | pre-nadir slope | 0.97 | 0.88 -1.07 | 0.51 |  |
|  | post-peak slope | 0.95 | 0.83 -1.09 | 0.47 |  | post-nadir slope | 1.06 | 0.98 -1.13 | 0.14 |  |
| **Model 2** | | | | | | | | | | |
| ER-ratio | peak | 1.02 | 0.97 -1.07 | 0.50 | 0.89 | nadir | 0.95 | 0.87 -1.04 | 0.23 | 0.69 |
|  | pre-peak slope | 1.01 | 0.88 -1.16 | 0.93 |  | pre-nadir slope | 0.96 | 0.87 -1.05 | 0.37 |  |
|  | post-peak slope | 0.99 | 0.87 -1.13 | 0.92 |  | post-nadir slope | 1.02 | 0.95 -1.10 | 0.52 |  |
| **Model 3** | | | | | | | | | | |
| O-ER-ratio | peak | 1.03 | 0.96 -1.09 | 0.45 | 0.81 | nadir | 0.91 | 0.81 -1.01 | 0.08 | 0.39 |
|  | pre-peak slope | 1.02 | 0.84 -1.23 | 0.85 |  | pre-nadir slope | 0.92 | 0.82 -1.03 | 0.16 |  |
|  | post-peak slope | 1.01 | 0.85 -1.21 | 0.90 |  | post-nadir slope | 1.06 | 0.97 -1.15 | 0.21 |  |

^a-f^ See footnotes to Table S8_1a

**Table S8_2b. Effects of ERI categorical exposures on morning and evening cortisol patterns. By exposure models 1 to 3.**

| **ERI categorical**  **exposures** | | **Morning cortisol**^a^  (n=5078/5978)^c^ | | | | | **Evening cortisol^b^**  (n=5285/6230)^c^ | | | | |
| --- | --- | --- | --- | --- | --- | --- | --- | --- | --- | --- | --- |
| **Exposure** | **Exposure category** | **Exposure effect**  **on**  **cortisol** | **Effect**  **Ratio**^d^ | **95% CI** | **p1**^e^ | **p2**^f^ | **Exposure effect**  **on**  **cortisol** | **Effect**  **Ratio**^d^ | **95% CI** | **p1**^e^ | **p2**^f^ |
| **Model 1** | | | | | | | | | | | |
| Effort | medium  vs  low | peak | 1.02 | 0.96 -1.08 | 0.46 | 0.80 | nadir | 0.98 | 0.89 -1.09 | 0.75 | 0.23 |
|  |  | pre-peak slope | 1.11 | 0.92 -1.35 | 0.28 |  | pre-nadir slope | 0.95 | 0.85 -1.06 | 0.34 |  |
|  |  | post-peak slope | 0.91 | 0.77 -1.07 | 0.25 |  | post-nadir slope | 0.94 | 0.86 -1.02 | 0.15 |  |
|  | high  vs  low | peak | 1.02 | 0.95 -1.10 | 0.57 |  | nadir | 1.03 | 0.91 -1.17 | 0.60 |  |
|  |  | pre-peak slope | 1.01 | 0.80 -1.26 | 0.95 |  | pre-nadir slope | 1.04 | 0.91 -1.19 | 0.59 |  |
|  |  | post-peak slope | 0.98 | 0.80 -1.19 | 0.81 |  | post-nadir slope | 0.97 | 0.87 -1.07 | 0.50 |  |
| Reward | medium  vs  high | peak | 0.99 | 0.93 -1.05 | 0.63 | 0.59 | nadir | 0.98 | 0.88 -1.08 | 0.69 | 0.80 |
|  |  | pre-peak slope | 0.93 | 0.77 -1.13 | 0.46 |  | pre-nadir slope | 1.00 | 0.90 -1.11 | 0.95 |  |
|  |  | post-peak slope | 1.06 | 0.90 -1.24 | 0.50 |  | post-nadir slope | 1.04 | 0.96 -1.13 | 0.32 |  |
|  | low  vs  high | peak | 0.91 | 0.83 -1.00 | 0.06 |  | nadir | 0.91 | 0.77 -1.06 | 0.23 |  |
|  |  | pre-peak slope | 0.87 | 0.66 -1.13 | 0.30 |  | pre-nadir slope | 0.94 | 0.79 -1.13 | 0.53 |  |
|  |  | post-peak slope | 1.25 | 0.96 -1.62 | 0.10 |  | post-nadir slope | 1.04 | 0.91 -1.19 | 0.57 |  |
| Overcom. | medium  vs  high | peak | 1.04 | 0.98 -1.10 | 0.17 | 0.19 | nadir | 0.98 | 0.88 -1.08 | 0.64 | 0.19 |
|  |  | pre-peak slope | 1.01 | 0.85 -1.21 | 0.88 |  | pre-nadir slope | 0.95 | 0.86 -1.06 | 0.38 |  |
|  |  | post-peak slope | 0.94 | 0.81 -1.09 | 0.42 |  | post-nadir slope | 1.05 | 0.97 -1.14 | 0.21 |  |
|  | low  vs  high | peak | 1.20 | 1.04 -1.40 | 0.01 |  | nadir | 0.73 | 0.56 -0.95 | 0.02 |  |
|  |  | pre-peak slope | 1.74 | 1.08 -2.80 | 0.02 |  | pre-nadir slope | 0.73 | 0.55 -0.97 | 0.03 |  |
|  |  | post-peak slope | 0.77 | 0.52 -1.13 | 0.19 |  | post-nadir slope | 1.14 | 0.94 -1.38 | 0.20 |  |

(continues on next page)

**Table S8_2b (continued). Effects of ERI categorical exposures on morning and evening cortisol patterns. By exposure models 1 to 3.**

| **ERI categorical**  **exposures** | | **Morning cortisol**^a^  (n=5078/5978)^c^ | | | | | **Evening cortisol^b^**  (n=5285/6230)^c^ | | | | |
| --- | --- | --- | --- | --- | --- | --- | --- | --- | --- | --- | --- |
| **Exposure** | **Exposure category** | **Exposure effect**  **on**  **cortisol** | **Effect**  **Ratio**^d^ | **95% CI** | **p1**^e^ | **p2**^f^ | **Exposure effect**  **on**  **cortisol** | **Effect**  **Ratio**^d^ | **95% CI** | **p1**^e^ | **p2**^f^ |
| **Model 2** | | | | | | | | | | | |
| ER-strain | low  vs  very low | peak | 0.96 | 0.88 -1.05 | 0.35 | 0.93 | nadir | 0.94 | 0.81 -1.10 | 0.45 | 0.67 |
|  |  | pre-peak slope | 0.98 | 0.74 -1.29 | 0.89 |  | pre-nadir slope | 0.91 | 0.77 -1.07 | 0.24 |  |
|  |  | post-peak slope | 1.13 | 0.90 -1.42 | 0.28 |  | post-nadir slope | 1.03 | 0.92 -1.16 | 0.58 |  |
|  | medium  vs  very low | peak | 1.03 | 0.96 -1.11 | 0.44 |  | nadir | 0.93 | 0.82 -1.06 | 0.28 |  |
|  |  | pre-peak slope | 1.03 | 0.83 -1.29 | 0.77 |  | pre-nadir slope | 0.92 | 0.80 -1.05 | 0.22 |  |
|  |  | post-peak slope | 1.00 | 0.82 -1.21 | 0.98 |  | post-nadir slope | 1.03 | 0.92 -1.14 | 0.63 |  |
|  | high  vs  very low | peak | 0.97 | 0.87 -1.09 | 0.65 |  | nadir | 0.93 | 0.77 -1.12 | 0.45 |  |
|  |  | pre-peak slope | 0.87 | 0.61 -1.22 | 0.41 |  | pre-nadir slope | 1.02 | 0.82 -1.27 | 0.88 |  |
|  |  | post-peak slope | 0.98 | 0.71 -1.35 | 0.89 |  | post-nadir slope | 1.12 | 0.95 -1.32 | 0.18 |  |
| **Model 3** | | | | | | | | | | | |
| O-ER-strain | low  vs  very low | peak | 1.02 | 0.90 -1.15 | 0.81 | 0.53 | nadir | 0.88 | 0.71 -1.09 | 0.25 | 0.39 |
|  |  | pre-peak slope | 1.31 | 0.84 -2.03 | 0.24 |  | pre-nadir slope | 0.82 | 0.65 -1.04 | 0.10 |  |
|  |  | post-peak slope | 0.93 | 0.70 -1.24 | 0.63 |  | post-nadir slope | 1.12 | 0.97 -1.31 | 0.12 |  |
|  | medium  vs  very low | peak | 1.09 | 0.99 -1.19 | 0.07 |  | nadir | 0.98 | 0.84 -1.14 | 0.83 |  |
|  |  | pre-peak slope | 1.22 | 0.95 -1.57 | 0.12 |  | pre-nadir slope | 0.93 | 0.79 -1.09 | 0.37 |  |
|  |  | post-peak slope | 0.93 | 0.74 -1.17 | 0.56 |  | post-nadir slope | 1.05 | 0.93 -1.19 | 0.44 |  |
|  | high  vs  very low | peak | 0.93 | 0.82 -1.06 | 0.30 |  | nadir | 0.85 | 0.69 -1.06 | 0.15 |  |
|  |  | pre-peak slope | 0.78 | 0.52 -1.15 | 0.21 |  | pre-nadir slope | 0.89 | 0.70 -1.13 | 0.33 |  |
|  |  | post-peak slope | 1.12 | 0.77 -1.64 | 0.56 |  | post-nadir slope | 1.20 | 1.00 -1.45 | 0.06 |  |

^a-f^ See footnotes to Table S8_1a

**Table S8_3a. Effects of DCS and ERI continuous exposures on the peak-to-nadir cortisol slope. By exposure models 1 to 3.**

| **DCS continuous exposures**^a^  (n=4969/5811)^b^ | | | | | **ERI continuous exposures**^a^  (n=4941/5811)^b^ | | | | |
| --- | --- | --- | --- | --- | --- | --- | --- | --- | --- |
|  | **Exposure unit**  **(score range)** | **Effect**  **Ratio**^c^ | **95% CI** | **p1**^d^ |  | **Exposure unit**  **(score range)** | **Effect**  **Ratio**^c^ | **95% CI** | **p1**^d^ |
| **Model 1** | | | | | | | | | |
| Demands | 1 unit (1-5) | 0.99 | 0.95 -1.03 | 0.55 | Effort | 1 unit (1-5) | 1.01 | 0.97 -1.04 | 0.71 |
| Control | 1 unit (1-5) | 1.05 | 0.99 -1.11 | 0.08 | Reward | 1 unit (1-5) | 1.06 | 0.99 -1.13 | 0.11 |
| Support | 1 unit (1-5) | 1.02 | 0.99 -1.06 | 0.16 | Overcom. | 1 unit (1-5) | 1.01 | 0.95 -1.07 | 0.81 |
| **Model 2** | | | | | | | | | |
| Job-strain | 1 unit (0.20-5) | 1.03 | 0.98- 1.09 | 0.21 | ER-ratio | 1 unit (0.20-5) | 1.04 | 0.98 -1.10 | 0.24 |
| **Model 3** | | | | | | | | | |
| Iso-strain | 1 unit (0.04-5) | 1.06 | 1.00- 1.13 | 0.05 | O-ER-ratio | 1 unit (0.04-5) | 1.05 | 0.97 -1.13 | 0.24 |

^a^ Adjusted for early- and late waking times, pre- and post-peak times, pre- and post-nadir times, and all other potential confounders.

^b^ Number of observations used in the analyses out of all observations with valid information on cortisol concentrations and relevant saliva sampling time information

^c^ Effect ratios are the ratios by which the peak-to-nadir ratio of cortisol concentrations change with a one unit change of continuous exposures or with a category level compared to the reference category.

Effect ratios above unity implies that the peak-to-nadir slope becomes steeper and vice versa.

^d^ p1: probability that the observed effect ratio differs from unity (test with one degree of freedom).

**Table S8_3b. Effects of DCS and ERI categorical exposures on the peak-to-nadir cortisol slope. By exposure models 1 to 3.**

| **DCS categorical exposures**^a^  (n=4969/5811)^b^ | | | | | | **ERI categorical exposures**^a^  (n=4941/5811)^b^ | | | | | |
| --- | --- | --- | --- | --- | --- | --- | --- | --- | --- | --- | --- |
| **Exposure** | **Exposure category** | **Effect**  **Ratio**^c^ | **95% CI** | **p1**^d^ | **p2**^e^ | **Exposure** | **Exposure category** | **Effect**  **Ratio**^c^ | **95% CI** | **p1**^d^ | **p2**^e^ |
| **Model 1** | | | | | | **Model 1** | | | | | |
| Demands | low | 1 |  |  | 0.57 | Effort | low | 1 |  |  | 0.88 |
|  | medium | 0.97 | 0.90 -1.03 | 0.31 |  |  | medium | 0.99 | 0.93 -1.06 | 0.84 |  |
|  | high | 0.99 | 0.91 -1.07 | 0.76 |  |  | high | 1.01 | 0.93 -1.10 | 0.75 |  |
| Control | low | 1 |  |  | 0.26 | Reward | high | 1 |  |  | 0.84 |
|  | medium | 1.05 | 0.99 -1.11 | 0.13 |  |  | medium | 1.02 | 0.95 -1.09 | 0.59 |  |
|  | high | 1.07 | 0.94 -1.23 | 0.31 |  |  | low | 1.02 | 0.92 -1.14 | 0.71 |  |
| Support | low | 1 |  |  | 0.09 | Overcom. | low | 1 |  |  | 0.54 |
|  | medium | 0.97 | 0.89 -1.05 | 0.41 |  |  | medium | 1.01 | 0.94 -1.08 | 0.81 |  |
|  | high | 1.20 | 1.00 -1.45 | 0.05 |  |  | high | 1.10 | 0.93 -1.30 | 0.27 |  |
| **Model 2** |  | | | | | **Model 2** | | | | | |
| Job-strain | very low | 1 |  |  | 0.21 | ER-ratio | very low | 1 |  |  | 0.73 |
|  | low | 0.99 | 0.92 -1.06 | 0.72 |  |  | low | 1.02 | 0.93 -1.13 | 0.62 |  |
|  | medium | 1.08 | 1.00 -1.17 | 0.06 |  |  | medium | 1.04 | 0.95 -1.13 | 0.38 |  |
|  | high | 1.07 | 0.85 -1.35 | 0.56 |  |  | high | 1.05 | 0.93 -1.20 | 0.43 |  |
| **Model 3** | | | | | | **Model 3** | | | | | |
| Iso-strain | very low | 1 |  |  | 0.53 | O-ER-ratio | very low | 1 |  |  | 0.85 |
|  | low | 1.03 | 0.90 -1.19 | 0.67 |  |  | low | 0.99 | 0.90 -1.10 | 0.91 |  |
|  | medium | 1.09 | 0.96 -1.24 | 0.19 |  |  | medium | 1.03 | 0.95 -1.12 | 0.48 |  |
|  | high | 1.12 | 0.80 -1.57 | 0.50 |  |  | high | 1.04 | 0.91 -1.18 | 0.57 |  |

^a-d^ See footnotes to Table S8_3a

^e^ p2: probability that estimated exposure level effects, considered together, differs from no effect. Degrees of freedom is the number of estimated effect ratios.
